# Are Mind-Body Exercise Beneficial for Treating Pain, Function, and Quality of Life in Middle-Aged and Old People With Chronic Pain? A Systematic Review and Meta-Analysis

**DOI:** 10.3389/fnagi.2022.921069

**Published:** 2022-06-21

**Authors:** Yu-Rong Wen, Jian Shi, Ya-Fei Wang, Yang-Yang Lin, Zheng-Yu Hu, You-Tian Lin, Xue-Qiang Wang, Yu-Ling Wang

**Affiliations:** ^1^Department of Sport Rehabilitation, Shanghai University of Sport, Shanghai, China; ^2^College of Kinesiology, Shenyang Sport University, Shenyang, China; ^3^Rehabilitation Medicine Center, The Sixth Affiliated Hospital of Sun Yat-sen University, Guangzhou, China; ^4^Postgraduate Research Institute, Guangzhou Sport University, Guangzhou, China; ^5^Department of Rehabilitation Medicine, Shanghai Shangti Orthopaedic Hospital, Shanghai, China

**Keywords:** mind-body exercises, chronic pain, old people, systematic review, meta-analysis

## Abstract

**Background:**

Aging is a significant risk factor in chronic pain development with extensive disability and greater health care costs. Mind-body exercise (MBE) has been scientifically proven to affect the pain intensity and physical health.

**Objectives:**

To assess the effects of MBE modes (Tai Chi, yoga, and qigong) for treating chronic pain among middle-aged and old people, compared with nonactive and active treatment, as well as function, quality of life, and adverse events.

**Methods:**

We searched PubMed, Embase, Web of Science, Cochrane Library, China National Knowledge Infrastructure (CNKI), Wanfang Database, and Chinese Scientific Journals Full-Text Database (VIP) till March 2022. No restrictions were chartered within the year and language of publication. We included randomized controlled trials of MBE treatment in middle-aged and elderly people with chronic pain. The overall certainty of evidence was evaluated by using the GRADE approach.

**Results:**

A total of 17 studies (*n* = 1,332) were included in this review. There was low-certainty evidence indicating that MBE had a moderate effect on reducing pain compared with the nonactive and active control group (standard mean difference (SMD): −0.64, 95% confidence interval (CI): −0.86 to −0.42, *P* < 0.001). Very-low-certainty evidence showed that the pooled SMD for the functional improvement was −0.75 (95% CI: −1.13 to −0.37, *P* < 0.001). Low-certainty evidence presented that no influence was observed in physical component summary (SMD: 0.23, 95% CI: −0.16 to 0.62, *P* = 0.24) and mental component summary (SMD: −0.01, 95% CI −0.39 to 0.36, *P* = 0.95).

**Conclusion:**

Our results indicated that MBE was an effective treatment for reducing symptoms of middle-aged and elderly people with chronic pain compared with nonactive and active control groups. TC and qigong had obvious benefits for knee osteoarthritis in self-reported function, but the efficacy of chronic low back pain was uncertain. No significant benefit of MBE on quality of life in older adults with chronic pain was found. More high-quality RCTs should be conducted to explore the efficacy and mechanism of MBE on chronic pain in middle-aged and elderly people from various dimensions, such as affective and cognitive dimensions.

**Systematic Review Registration:**

https://www.crd.york.ac.uk/PROSPERO/display_record.php?RecordID=316591, identifier CRD42022316591.

## Introduction

Pain is currently defined by the International Association for the Study of Pain as “an unpleasant sensory and emotional experience associated with or resembling that associated with actual or potential tissue damage” (Raja et al., 2020). Chronic pain is pain that lasts 12 weeks or longer (Treede et al., 2019). In the United States alone, more than one in five adults experience chronic pain at an estimated cost of 560 billion dollars a year (Reuben et al., 2015; Yong et al., 2022).

Aging is a significant factor in the development of chronic pain (Tsang et al., 2008). With the gradually increasing trend of global aging, the prevalence of chronic pain is increasing in middle-aged and old people and has a connection with extensive disability and greater health care costs (Cho et al., 2015; Reid et al., 2015; Corsi et al., 2018; Sun et al., 2019; Li et al., 2021). One survey based on Chinese people revealed the prevalence of knee osteoarthritis (KOA) increased with age, surging after 55 years old (Chen et al., 2021). Low back pain (LBP), a leading risk factor for physical disability worldwide, affects nearly 20–25% of the population older than 65 years around the world (Vadalà et al., 2020). However, the clinical efficacy and side effects of pharmacologic treatment for elderly people with chronic pain as well as the potential effects are unclear (McLachlan et al., 2011; Al-Qurain et al., 2020). Hence, identifying effective nonpharmacological approaches for middle-aged and elderly people with chronic pain is urgent.

Over the past decades, complementary and alternative medicine (CAM) has become popular among patients with various medical conditions (Weeks, 2016). Mind-body exercise (MBE) has been included in categories of CAM practices. The United States established a professional National Center for Complementary and Alternative Medicine to train professional CAM researchers, conduct research on the rigorous scientific role of MBE, and communicate authoritative information to the public. With the advancement of research techniques, more forms of MBE have been scientifically proven to affect neural activity and physical health (Clarke et al., 2015). Tai Chi (TC), yoga, and qigong (e.g., Baduanjin and Wuqinxi), the three most popular MBE modes, involve a variety of movements, such as postures with stretching and relaxation of skeletal muscles, breath control, and meditative state of mind (Bower and Irwin, 2016; Zou et al., 2018). They have been used as a treatment for different chronic pain conditions (Teut et al., 2016; Wang et al., 2016, 2018; Liu et al., 2019) and are considered suitable for middle-aged and old people (Reid et al., 2015; Siu et al., 2021). However, results of these studies are mixed. For example, a previous study has shown that TC may effectively improve pain intensity in middle-aged and elderly patients with chronic LBP (CLBP) (Liu et al., 2019), but studies about other MBE modes have not shown similar results (Teut et al., 2016). Natalia et al. published a structured review in similar areas (Morone and Greco, 2007), which included the study lacked randomized controlled trials (RCTs). Conclusions about the efficacy of MBE for chronic pain in elderly people must be tentative. No systematic review had tried to investigate the effects of MBE on middle-aged and elderly people with chronic pain to obtain a deeper awareness of it as a bona fide CAM therapy for chronic pain. Only a few reviews have paid attention to the role of MBE in the treatment of adults with chronic pain (Lauche et al., 2013; Bai et al., 2015; Kong et al., 2016; Hall et al., 2017; Wieland et al., 2017; Zou et al., 2019), and most of these studies either focused on a single chronic pain condition (Lauche et al., 2013; Wieland et al., 2017; Zou et al., 2019) or investigated a single MBE mode (Bai et al., 2015; Kong et al., 2016; Hall et al., 2017). Due to language barriers and limited retrieval resources, most reviews did not include Chinese RCTs (Lauche et al., 2013; Hall et al., 2017; Wieland et al., 2017, Zou et al., 2019).

Thus, this study aimed to represent the first systematic review and meta-analysis of current evidence from RCTs to ascertain the effectiveness of the three most popular MBE modes (i.e., TC, yoga, and qigong) among middle-aged and elderly people with chronic pain. Moreover, we investigated the effects of MBE on quality of life (QOL) and subjective physical function. We hypothesized that MBE intervention may benefit pain, physical function, and QOL in middle-aged and elderly with chronic pain, and compared with qigong and yoga, TC may have a better therapeutic effect. Our study raised the level of evidence by performing a secondary analysis of published RCTs in this area representing an important addition to the literature. Results provided robust evidence and guidance to clinicians in the use of CAM for middle-aged and old people with chronic pain and better manage patients' expectations.

## Methods

### Protocol and Registration

We prospectively registered the protocol in the PROSPERO database with registration number CRD42022316591. This systematic review and meta-analysis were reported in line with the PRISMA guidelines and are shown in Supplementary Material 1.

### Search Strategy

PubMed, Embase, Web of Science, Cochrane Library, China National Knowledge Infrastructure (CNKI), Wanfang Database, and Chinese Scientific Journals Full-Text Database (VIP) were confined to search to define eligible studies from the first data available in March 2022. No restrictions were chartered within the year and language of publication. The following key words are searched: “mind-body exercise,” “tai chi,” “yoga,” “qigong,” “aged,” “middle-aged and old people,” “old people,” and “chronic pain.” Complete search strategies are shown in Supplementary Material 2.

### Eligibility Criteria

#### Inclusion Criteria

· Design of studies: parallel or crossover RCTs.
· Subjects: middle-aged and older patients (aged ≥ 45 years) with chronic pain (duration of pain ≥ 12 weeks).
· Types of intervention: interventions were the prescription of MBE training alone, including tai chi, yoga, and qigong, without additional treatments (e.g., pharmacotherapy, manipulation, or cognitive behavior therapy).
· Types of control group: control group of any form (e.g., waitlist, physical exercise, and education) other than MBE.
· Outcomes: main outcomes related to the intensity of pain were measured after intervention. Secondary outcomes include subjective physical function and QOL.
· Published form of article: journal articles, theses, and dissertations.
· Studies must contain raw data of interest outcomes or can be extracted from figures and tables.

#### Exclusion Criteria

· Studies unable to satisfy the inclusion criteria.
· Studies that were published in the form of conference abstracts, researcher protocol, and books.

### Study Selection

Two assessors (i.e., YRW and JS) perform a preliminary screening of all retrieved articles based on title and abstract. If the topic of studies cannot be defined by the title and abstract, the full text of the article shall be evaluated. Any disaccords should be resolved through discussion to reach consensus. When the two assessors cannot agree, the corresponding author (YLW) will evaluate the study to determine whether it meets the inclusion criteria of the review.

### Data Extraction

Tables' data extraction for each selected study was completed independently by two evaluators (YTL and ZYH) using a standard information extraction form, which was developed jointly by them. Two corresponding authors (YLW and XQW) reviewed the extracted data, including the publication information (e.g., author, year, and country of origin), subject characteristics (e.g., age, gender, sample size, and pain conditions), study design (e.g., parallel or crossover trail, two-arm or multiarm parallel trial), intervention and control groups (e.g., MBE modes, duration weeks, and follow-up weeks), and adverse events.

The mean and standard deviation (mean ± SD) of primary and secondary outcomes of preintervention and post intervention for the MBE groups and control groups were directly drawn from published data of the studies. When data were reported at more than one time point, we used only the data immediately at the end of the intervention. If the outcome was expressed only as a graph, the software Engauge Digitizer 10.8 (Mitchell et al., 2017) was used to extract the required data. When raw data cannot be sufficiently extracted, we contacted the authors of these studies to provide it; the RevMan 5.3 calculator was used to convert them to means and SDs when the standard errors (SEs), confidence intervals (Cls), or interquartile ranges (IQRs) were supplied rather than means and SDs.

### Risk of Bias and GRADE

Two authors (YRW and JS) independently evaluated the quality of methods and the risk of bias of these studies by the Cochrane Risk of Bias Tool (Higgins et al., 2016), which divided the quality risk into three categories, namely, low, high, and unclear, which examined potential selection bias, performance bias, detection bias, attrition bias, reporting bias, and other bias. Given the exercise interventions involved in these studies, it is unclear whether blind subjects to treatment allocation are successful; therefore, patients blinding was considered as unclear risk of bias for each study. Furthermore, we assessed the quality of evidence outcomes using the recommendations assessment, development, and evaluation (GRADE) pathway (Atkins et al., 2004). GRADE may reduce the quality of evidence in the systematic evaluation of intervention: inconsistency, risk of bias, inaccuracy, indirectness, and publication bias. GRADEpro will be used to evaluate the five factors and classify the quality of evidence into four grades, namely, high quality, medium quality, low quality, and very low quality.

### Data Synthesis and Analysis

Meta-analysis was generated by exploring the STATA/MP 16.0 software (StataCorp, Texas, USA) with the metan command. The standard mean difference (SMD) of the change score (end-point minus baseline score) and its 95% CI were used for assessing the effect size (ES) of MBE and control. We used *P*-value and I^2^ to evaluate the heterogeneity. If *P* ≥ 0.05 and I^2^ ≤ 50%, the heterogeneity among studies was small, and the fixed effect model was adopted. If *P* < 0.05 and I^2^ > 50%, it shows that there is heterogeneity among studies, and the random effect model is adopted.

The size of heterogeneity was deciphered on the base of Cochrane Collaboration: I^2^ values of low heterogeneity (I^2^ < 25%), moderate heterogeneity (I^2^ < 50%), and high heterogeneity (I^2^ > 75%) (Higgins et al., 2003). *P* < 0.05 was considered that the difference was statistically significant. Publication bias was evaluated by a funnel plot. The Egger test was used to assess whether the degree of asymmetry was significant. The funnel asymmetry due to publication bias will be adjusted using the trim and fill method.

Four subgroup analyses were conducted to explore factors impacting the result of MBE on chronic pain: chronic pain conditions (KOA vs. CLBP vs. other pain [neck pain and chronic multisite pain]), number of sessions (1–15 sessions vs. 15–30 sessions vs. 30–45 sessions vs. more than 45 sessions), MBE modes (TC vs. yoga vs. qigong), and type of control (active control and nonactive control).

## Results

### Search Results

**Figure 1:**
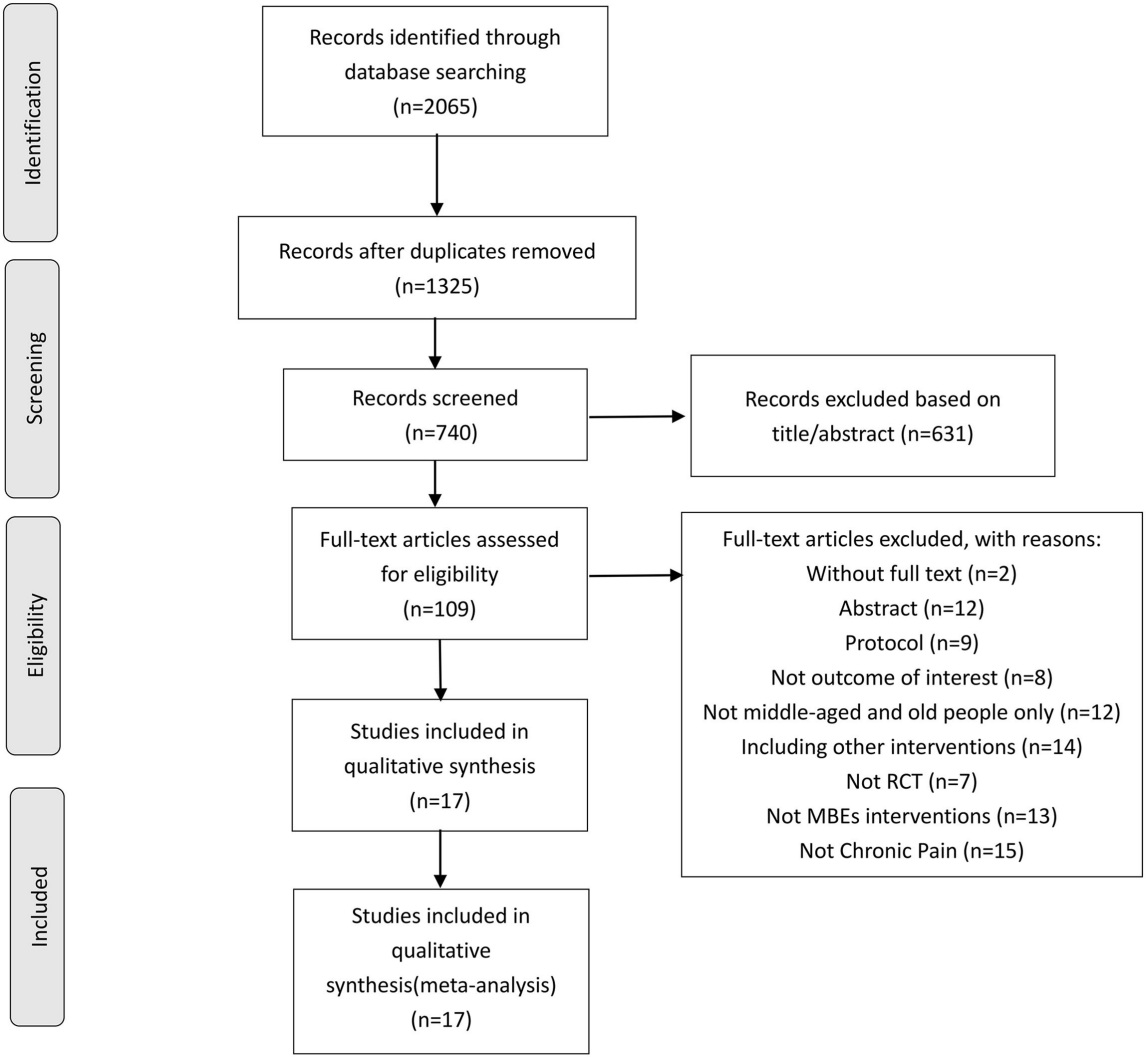
Study selection flowchart according to the PRISMA guidelines, Preferred Reporting Items for Systematic Reviews and Meta-Analyses. RCT, randomized controlled trials.

The process of study screening is shown in Figure 1. A total of 2,065 potential studies were identified by preliminary search for seven databases of which 1,325 duplicate studies were excluded. A total of 631 studies were excluded by title and abstract judgment, which are unable to fulfill the inclusion criteria of this systematic review. Then, we judged the full text of the remaining 109 studies, and 92 of them were excluded for several reasons including not RCTs (*n* = 7), not chronic pain (*n* = 15), abstract (*n* = 12), study protocol (*n* = 9), not middle-aged and elderly people only (*n* = 12), full-text not available (*n* = 2), not MBE interventions (*n* = 13), not outcome of interest (*n* = 8), including other interventions (*n* = 14). In this study, 17 eligible RCTs were included in this systematic review, and all of them were included in quantitative synthesis.

### Characteristics of the Included Studies

Table 1 summarizes all 17 reviewed MBE RCTs (*n* = 1,481). The sample size of a single article ranged from 26 to 204 participants (the average age range is 55–76 years). Almost all studies included both women and men, except (Cheung et al., 2017) which did not report sex ratios and (Qiu, 2018) which included only women. A total of 11 studies (64.7%) (Raub, 2002; Cheung et al., 2014, 2017; Wang et al., 2016, 2021; Hu, 2017; Song et al., 2017; Cao, 2018; Qiu, 2018; Ye et al., 2019; Xiao et al., 2020) included patients who suffer chronic pain from KOA. Three studies (Teut et al., 2016; Cao, 2018; Liu et al., 2019) included patients with CLBP and three studies (Yang et al., 2005; von Trott et al., 2009; You et al., 2018) included other chronic pain patients.

**Table 1:** Principal characteristics of included studies.

**Author, Year**	**Country**	**Sample size (F/M)**	**Age, Mean ±SD (years)**	**Pain conditions**	**Duration weeks**	**Follow-up** **weeks**	**Main pain outcome assessments**	**Experimental group intervention** **(T/session)**	**Control group intervention**	**Adverse events**
Brismée et al. (2007)	USA	34/7	69.94 ± 9.33	KOA	12	18	VAS/WOMAC	Yang-style Tai Chi (40 min/3 times a week/12 weeks/36 sessions)	Attention control	NO
Fransen et al. (2007)	Australia	112/40	70.06 ± 6.18	HOA/KOA	12	NO	WOMAC/SF-12	Sun-style Tai Chi (60 min/2 times a week/12 weeks/24 sessions)	Waiting list	NO
von Trott et al. (2009)	Germany	111/6	75.82 ± 7.43	NP	12	24	VAS/NPAD	Dantian Qigong (45 min/2 times a week/12 weeks/24 sessions)	Waiting list	Nausea/Aching muscles/Muscle tension
Yang et al. (2005)	Korea	32/8	72.62 ± 6.5	Chronic pain	4	6	VAS	Korean Qigong (20 min/2 times a week/4 weeks/8 sessions)	Waiting list	NA
Cheung et al. (2014)	USA	36/0	71.9 ± 6.04	KOA	8	12	WOMAC/SF-12	Hatha Yoga (60 min/once a week/8 weeks/8 sessions)	Waiting list	NO
Ding (2014)	China	20/20	61.25 ± 4.73	LBP	12	NO	VAS	Baduanjin Qigong (40 min/5 times a week/12 weeks/60 sessions)	Pharmacological treatment	NA
Cheung et al. (2017)	USA	70/13	71.62 ± 7.94	KOA	8	NO	VAS/WOMAC/SF-12	Hatha Yoga (45 min/once a week/8 weeks/8 sessions)	Education	NO
Teut et al. (2016)	Germany	156/20	72.65 ± 5.63	LBP	12	24	VAS/FFbHR	Dantian Qigong (90 min/once a week/12 weeks/12 sessions) Vini Yoga (45 min/2 times a week/12 weeks/24 sessions)	Waiting list	NA
Wang et al. (2016)	USA	143/61	60.2 ± 10.47	KOA	12	24/52	WOMAC/SF-36	Yang-style Tai Chi (60 min/2 times a week/12 weeks/24 sessions)	Physical therapy	NO
Cao (2018)	China	34/7	69.96 ± 9.33	KOA	12	18	WOMAC	Tai Chi (60 min/3 times a week/12 weeks/36 sessions)	Education	NO
Hu (2017)	China	108/32	58.8 ± 7.67	KOA	12	NO	VAS/WOMAC	Tai Chi (60min/5 times a week/12 weeks/60 sessions) Baduanjin Qigong (60 min/5 times a week/12 weeks/60 sessions)	Education	NO
Qiu (2018)	China	62/0	56.08 ± 3.75	KOA	12	NO	VAS/WOMAC	Baduanjin Qigong (30min/3 times a week/12 weeks/36 sessions)	Pharmacological treatment	NA
You et al. (2018)	USA	35/10	74.53 ± 8	Chronic multisite pain	12	NO	BPI	Yang-style Tai Chi (60 min/2 times a week/12 weeks/24 sessions)	Physical exercise	NO
Liu et al. (2019)	China	32/11	59.38 ± 4.26	LBP	8	NO	VAS	Chen-style Tai Chi (60 min/3 times a week/12 weeks/36 sessions)	No treatment	NA
Xiao et al. (2020)	China	62/37	70.4 ± 9.72	KOA	24	NO	WOMAC	Wuqinxi Qigong (60 min/4 times a week/24 weeks/96 sessions)	Physical therapy	NO
Ye et al. (2019)	China	30/20	63.78 ± 6.07	KOA	12	NO	WOMAC	Baduanjin Qigong (40 min/3 times a week/12 weeks/36 sessions)	Waiting list	NO
Wang et al. (2021)	China	69/15	65.23 ± 3.2	KOA	24	NO	WOMAC/SF-12	Baduanjin Qigong (40 min/3 times a week/24 weeks/72 sessions)	QSE	NO

*M, male; F, female; LBP, low back pain; NP, neck pain; KOA, knee osteoarthritis; HOA, hip osteoarthritis; QSE, quadriceps strengthening exercises; CSE, core stabilization exercise; VAS, visual analog scale; BPI, Brief Pain Inventory; WOMAC, Western Ontario and McMaster Universities Osteoarthritis Index; NPAD, Neck Pain and Disability; SF-36, Health-Related Short Form 36; SF-12, Health-Related Short Form 12; FFBH-R, Hanoverian Functional Questionnaire for Routine Diagnosis Of Dysfunction Due To Back Pain; NA, not available*.

The researchers implemented various mindful exercise interventions, which included yoga [two Hatha yoga (Cheung et al., 2014, 2017) and one Viniyoga (Teut et al., 2016)], TC [two Yang-style TC (Wang et al., 2016; Song et al., 2017); one Chen-style TC (Liu et al., 2019); one Sun-style TC (Raub, 2002); and three just demonstrated TC (Hu, 2017; Cao, 2018; You et al., 2018)], and qigong [five Baduanjin qigong (Ding, 2014; Hu, 2017; Qiu, 2018; Ye et al., 2019; Wang et al., 2021); one Wu Qin Xi Qigong (Xiao et al., 2020); one Korean qigong (Yang et al., 2005); and two just mentioned qigong (von Trott et al., 2009; Teut et al., 2016)]. There were five control comparators included nonphysical therapy [usual care (Cheung et al., 2014; Ding, 2014; Teut et al., 2016; Qiu, 2018; Ye et al., 2019); wait list (Raub, 2002; Yang et al., 2005; von Trott et al., 2009); education (Cheung et al., 2017; Hu, 2017); attention control group (Song et al., 2017)], physical therapy (Wang et al., 2016; You et al., 2018; Xiao et al., 2020), quadriceps strengthening exercises (QSEs) (Wang et al., 2021), core stabilization (Liu et al., 2019), and stretching (Cao, 2018). The intervention duration of all mindful exercises was between 4 and 24 weeks, and sessions ranged from 8 to 96 weeks. The frequency of the intervention group varied greatly, ranging from one to five times a week. The length of each exercise class also varied, ranging from 20 to 90 min a class. Eight studies were followed up after the intervention for 6–52 weeks.

Pain intensity as the main outcome can be obtained from 17 studies. The evaluation scales were Western Ontario and McMaster Universities Osteoarthritis Index (WOMAC)-pain (Raub, 2002; Cheung et al., 2014; Wang et al., 2016, 2021; Hu, 2017; Ye et al., 2019; Xiao et al., 2020) and Visual Analog Scale (VAS) (Yang et al., 2005; von Trott et al., 2009; Teut et al., 2016; Cheung et al., 2017; Song et al., 2017; Cao, 2018; Qiu, 2018; You et al., 2018; Liu et al., 2019). Twelve studies reported functional improvement outcomes using WOMAC-function (Cheung et al., 2014, 2017; Ding, 2014; Wang et al., 2016, 2021; Hu, 2017; Song et al., 2017; Qiu, 2018; Ye et al., 2019; Xiao et al., 2020), FFbHR (Teut et al., 2016), and Neck Pain and Disability (NPAD) (von Trott et al., 2009). QOL indicators were obtained from eight studies using the Health-related Short Form 12 (SF-12) (Raub, 2002; Cheung et al., 2014, 2017; Wang et al., 2021) and Health-related Short Form 36 (SF-36) scales (von Trott et al., 2009; Teut et al., 2016; Wang et al., 2016).

### Effects of the Intervention

#### Primary Outcomes: Pain Intensity

**Figure 2:**
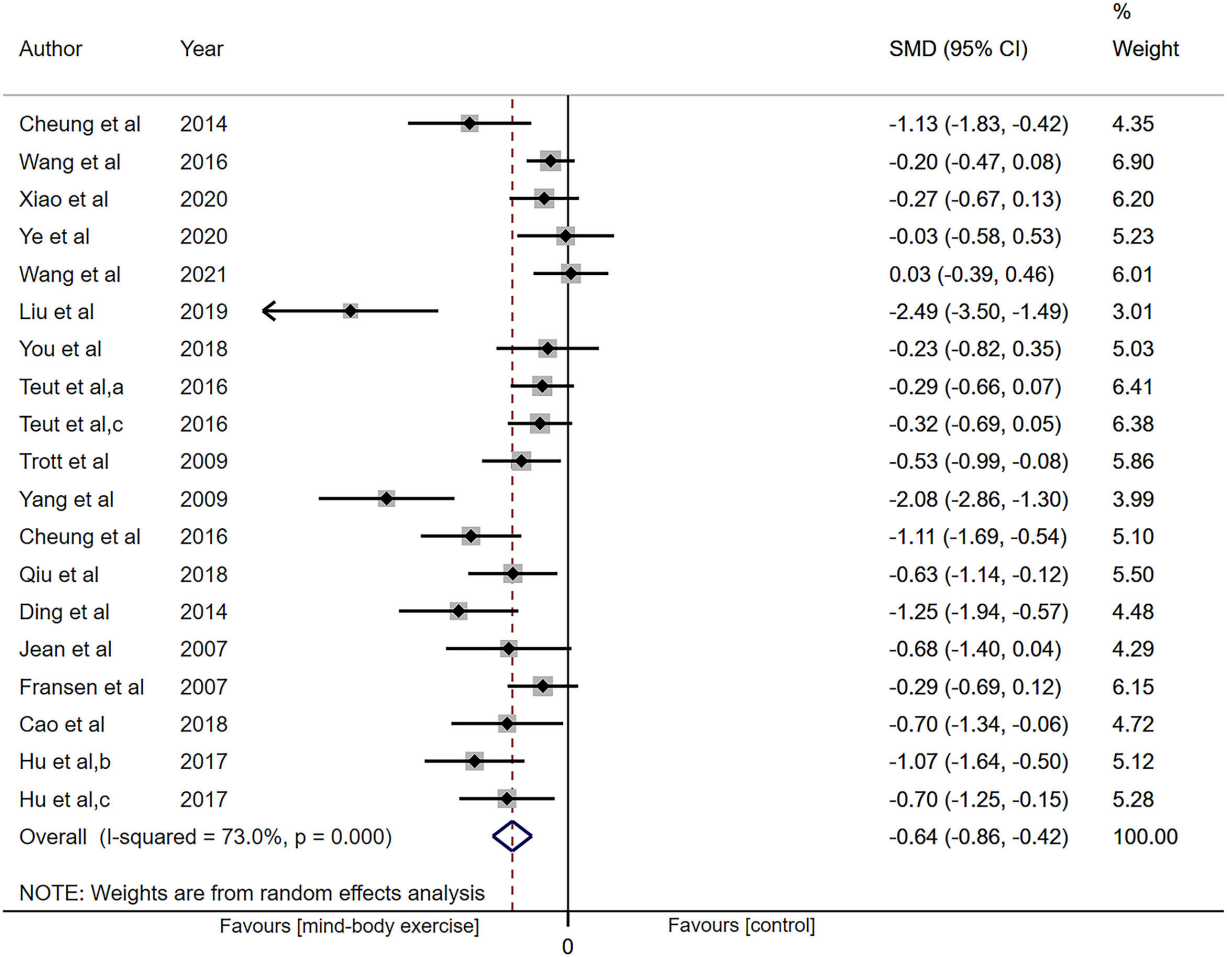
Forest plot showing overall effect sizes (Hedges' g) of mind-body exercises on pain intensity within studies. These plots show the pooled standard mean difference (SMD) (large diamond shape) and I^2^ resulting from the meta-analysis.

All 17 articles included in this study (*n* = 1,332) measured pain intensity. Low-certainty evidence showed that MBE had a moderate effect on reducing pain compared with both the nonactive and active control group (SMD: −0.64, 95% CI: −0.86 to −0.42, *P* < 0.001; Figure 2), but the heterogeneity was relatively high (I^2^ = 73.0%, *P* < 0.001).

Subgrouping analysis by painful conditions significantly decreased heterogeneity in the KOA subgroup (SMD: −0.51, 95% CI: −0.73 to −0.28, *P* < 0.001, I^2^ = 59.1%, *P* = 0.005; Supplementary Figure S1), and increased the ES in CLBP subgroup (SMD: −0.95, 95% CI: −1.66 to −0.24, *P* = 0.008) with heterogeneity of 86.2% (*P* < 0.001). But in other chronic pain subgroups, it did not show the ES (SMD: −0.91, 95% CI: −1.85 to 0.03, *P* = 0.06) with heterogeneity of 86.7% (*P* = 0.001).

Categorizing studies by intervention methods of experimental group, the results show that three different MBE still had a good effect on reducing pain but did not cut heterogeneity observably. The overall data from 3 trials demonstrated that yoga had more analgesic effect compared with the control group (SMD: −0.80, 95% CI: −1.41 to −0.18, *P* = 0.01, I^2^ = 74.6%; Supplementary Figure S2). The TC subgroup revealed an effect (SMD: −0.69, 95% CI: −1.10 to −0.27, *P* = 0.001, I^2^ = 76.6%). In the qigong subgroup, the results synthesized from nine trials show ES (SMD: −0.57, 95% CI: −0.91 to −0.24, *P* = 0.001, I^2^ = 75.0%).

Categorizing studies by number of sessions (≤ 15 sessions vs. 15–30 sessions vs. 30–45 sessions vs. > 45 sessions). The ES of subgroup in ≤ 15 sessions (SMD: −1.12, 95% CI: −2.09 to −0.16, *P* = 0.02) and 30–45 sessions (SMD: −0.86, 95% CI: −1.39 to −0.32, *P* = 0.002) increased. While subgrouping analysis decreased the ES (SMD: −0.29, 95% CI: −0.46 to −0.12, *P* = 0.001) and decreased heterogeneity (I^2^ = 0.0%, *P* = 0.81) in fifteen to thirty sessions subgroup (Supplementary Figure S3).

#### Secondary Outcomes: Physical Function

**Figure 3:**
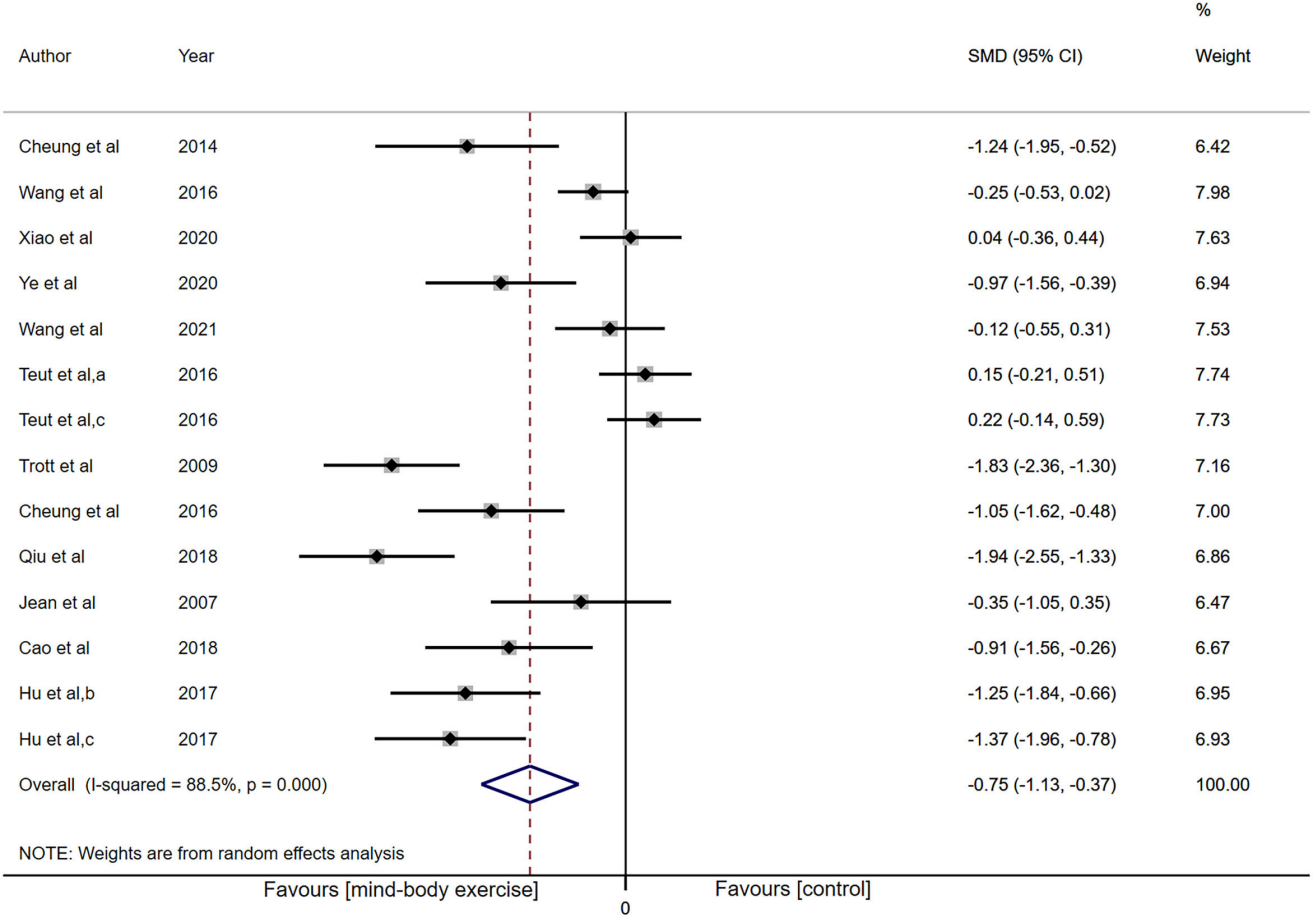
Forest plot showing overall effect sizes (Hedges' g) of mind-body exercises on self-reported function within studies. These plots show the pooled SMD (large diamond shape) and I^2^ resulting from the meta-analysis.

Adequate information was available from 12 studies (*n* = 1,082) for functional improvement analysis. Low-certainty evidence has shown that the pooled SMD for the result was −0.75 (95% CI: −1.13 to −0.37, *P* < 0.001; Figure 3) with the heterogeneity of 88.5% (*P* < 0.001).

Subgrouping studies by type of painful condition, no evidence of an effect of MBE on CLBP was observed (SMD: 0.19, 95% CI: −0.07 to 0.44, *P* = 0.16), but its heterogeneity significantly diminished (I^2^ = 0%, *P* = 0.79; Supplementary Figure S4). The MBE demonstrated a noteworthy analgesic effect on KOA (SMD: −0.83, 95% CI: −1.20 to −0.45, *P* < 0.001) with the heterogeneity of 83.1%. In other pain subgroup (neck pain), there was an increased effect (SMD: −1.83, 95% CI: −2.36 to −1.30, *P* < 0.001) with the heterogeneity of 0%.

Categorizing studies by intervention methods of experimental group, there was no indication of an effect of yoga treatment (SMD: −0.68, 95% CI: −1.64 to 0.28, *P* = 0.17; Supplementary Figure S5), while the TC group (SMD: −0.66, 95% CI: −1.17 to −0.15, *P* = 0.01) and qigong group (SMD: −0.83, 95% CI: −1.50 to −0.16, *P* = 0.02) displayed a significant analgesic effect.

Categorizing studies by number of sessions (≤ 15 sessions vs. 15–30 sessions vs. 30–45 sessions vs. > 45 sessions). Subgroup of 30–45 sessions showed an increased effect (SMD: −1.09, 95% CI: −1.61 to −0.58, *P* < 0.001) with decreased heterogeneity (I^2^ = 67.7%, *P* = 0.02), but there was no indication of an effect in other subgroups (Supplementary Figure S6).

#### Secondary Outcomes: QOL

Physical component summary (PCS) and mental component summary (MCS), as the two parts of QOL (i.e., SF-36 and SF-12), reflect various dimensions of QOL.

##### Physical Component Summary

**Figure 4:**
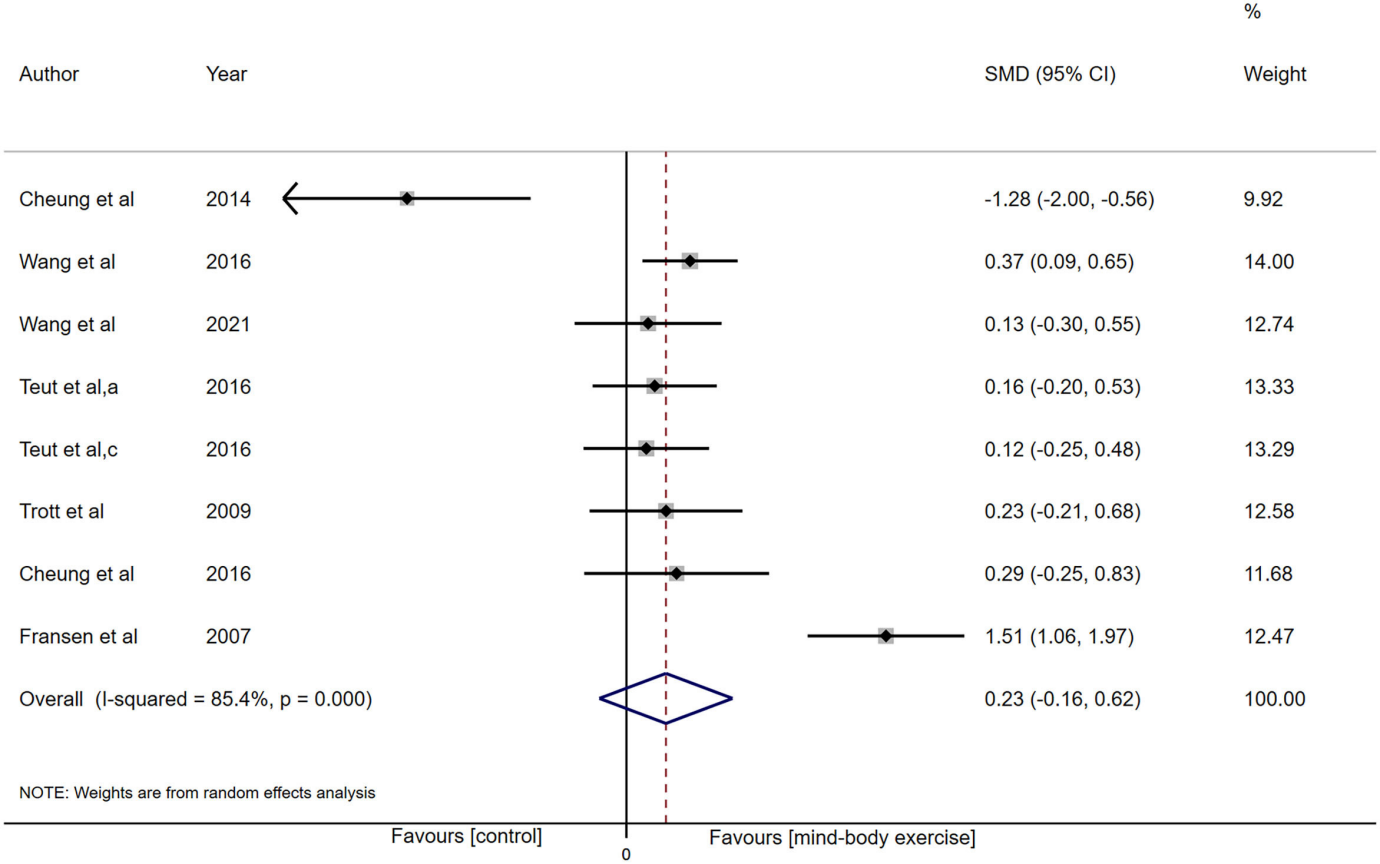
Forest plot showing overall effect sizes (Hedges' g) of mind-body exercises on physical component summary within studies. These plots show the pooled SMD (large diamond shape) and I^2^ resulting from the meta-analysis.

Sufficient data were accessible from 8 studies (*n* = 787) for PCS analysis. The synthesized result showed heterogeneity (I^2^ = 85.4%, *P* < 0.001), and low-certainty evidence of the ES was not in favor of that MBE can improve PCS (SMD: 0.23, 95% CI: −0.16 to 0.62, *P* = 0.24; Figure 4).

##### Mental Component Summary

**Figure 5:**
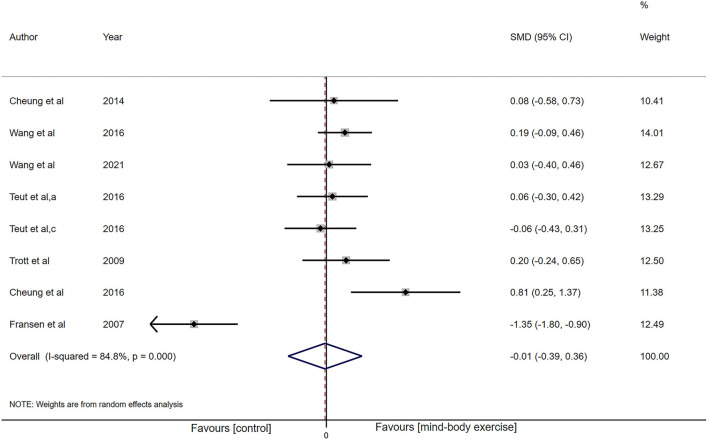
Forest plot showing overall effect sizes (Hedges' g) of mind-body exercises on mental component summary within studies. These plots show the pooled SMD (large diamond shape) and I^2^ resulting from the meta-analysis.

In terms of the MCS, eight investigations (*n* = 787) provided data for MCS analysis. There was high heterogeneity (I^2^ = 84.8%, *P* < 0.001), while low-certainty evidence presented there was no influence (SMD: −0.01, 95% CI −0.39 to 0.36, *P* = 0.95; Figure 5).

### Risk of Bias and GRADE

**Figure 6:**
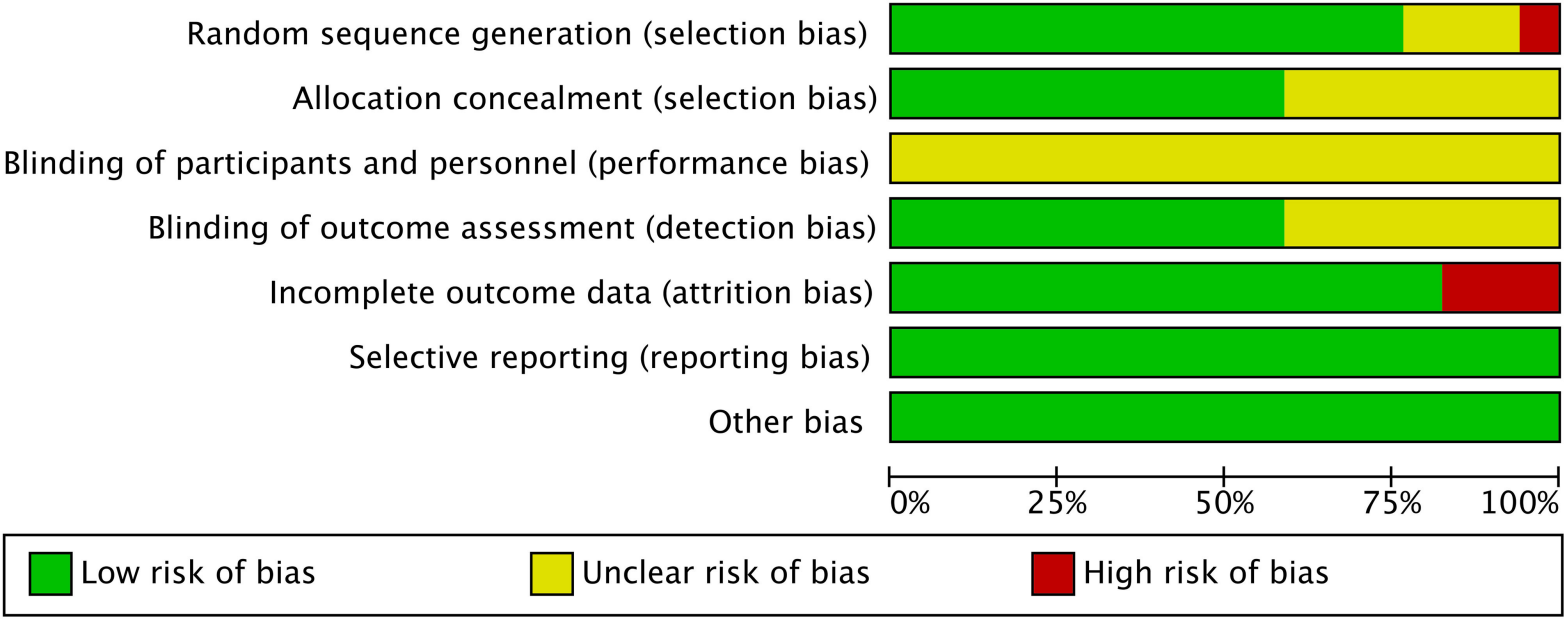
Risk of bias graph: review authors' judgments about each risk of bias item presented as percentages across all included studies.

**Figure 7:**
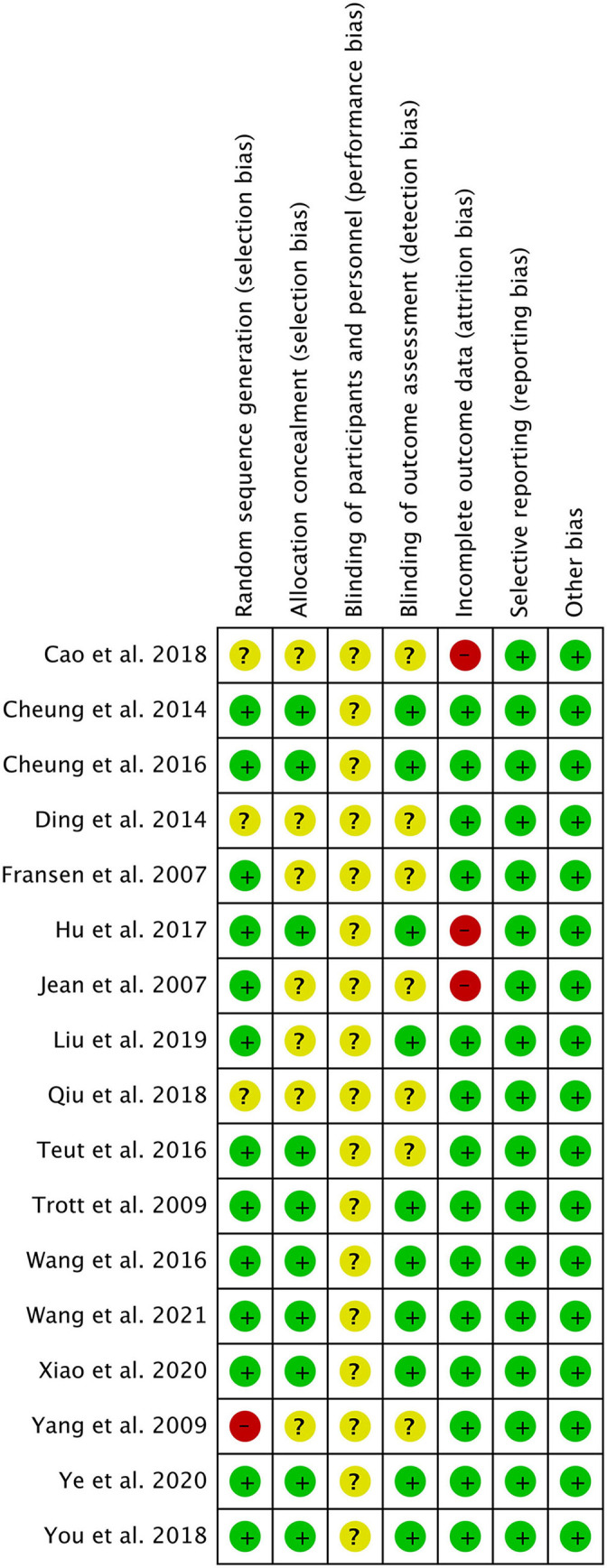
Methodological quality summary: review authors' judgments about each methodological quality item for each included study.

The results of the bias risk graph included in the study are shown in Figures 6, 7. Most articles showed low-risk random sequence generation (76%), allocation concealment (58%), incomplete outcome data (82%), selective reporting (100%), and other bias (100%). Due to the particularity of the experimental intervention protocol design, the low risk of blinding of participants and personnel ratio is 0%, and 58% of the studies showed low risk in the aspect of blinding of outcome assessment. The quality of evidence assessed by the GRADE approach is shown in Supplementary Material 3.

### Sensitivity Analysis

Removing the literature comparisons one by one did not significantly change the heterogeneity of pain outcomes. This indicated that the outcomes achieved were strong and reliable. No outliers were found because the total ES of each investigation was within 2 SD of the total average impact size. Additionally, four high-risk biased trials were excluded to determine whether they could influence the outcome of the meta-analysis. The other 13 studies maintained statistically meaningful in an effect estimate of −0.51 (95% CI: −0.74 to −0.28) with heterogeneity of 69.7%. There was less difference between the results before and after analysis, and the heterogeneity was uninfluenced.

### Publication Bias

**Figure 8:**
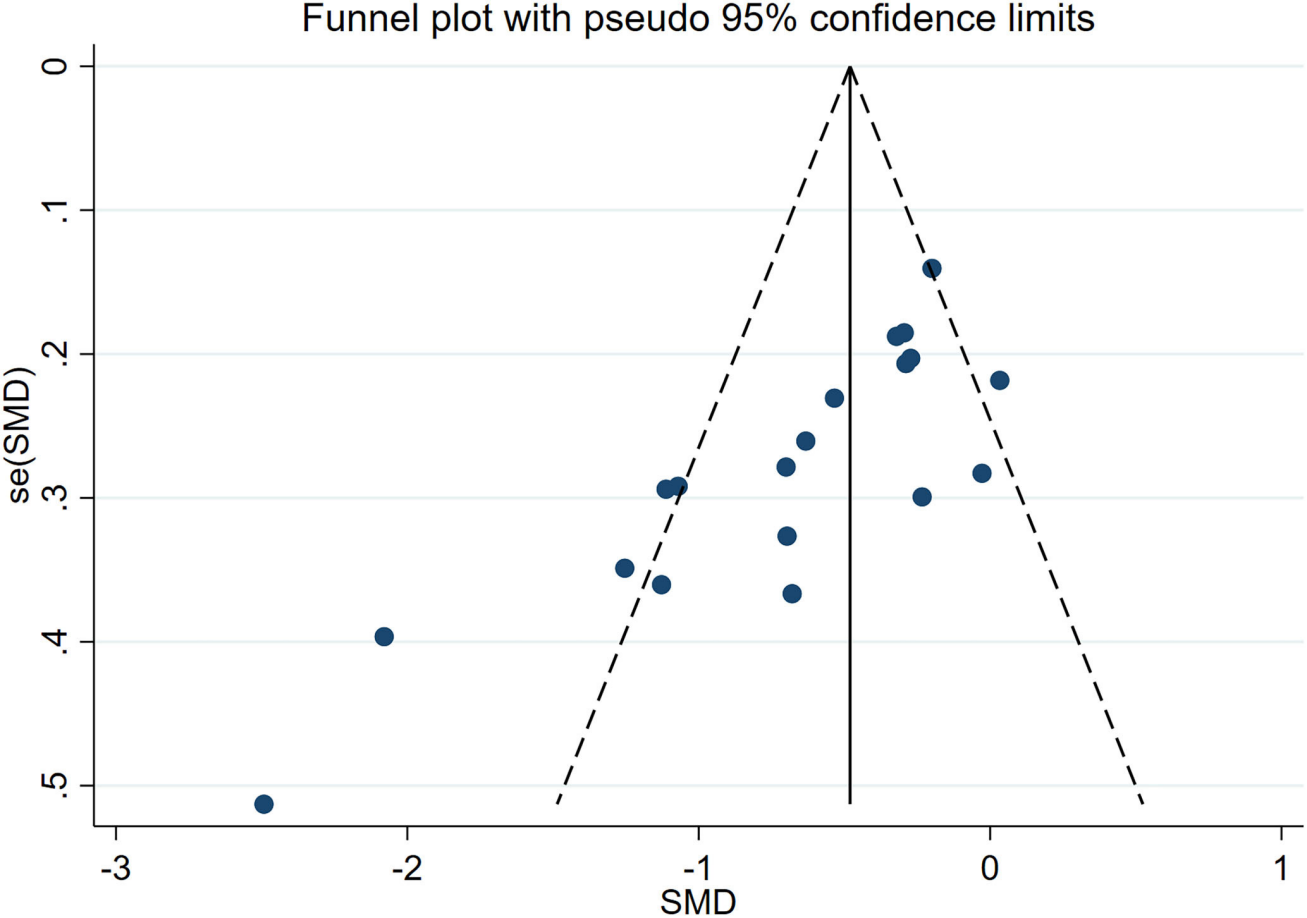
Funnel plots with standard errors plotted against effect sizes for determining publication bias in chronic pain intensity.

The Egger's test results show physical function scores (*P* = 0.004, Supplementary Figure S7) and pain intensity scores (*P* = 0.001, Figure 8) observed significant publication bias, and we corrected the results by trim and fill analysis. The pooled estimate and 95% CI of pain intensity scores and physical function scores calculated by the fixed-effect model and random-effect model were −0.481 (−0.592, −0.37) and −0.639 (−0.862, −0.415), −0.49 (−0.615, −0.365) and −0.747 (−1.126, −0.368), respectively, after trim and fill analysis. Their results did not change before and after the trim and fill analysis, showing our results were robust.

### Adverse Event of Intervention

Among the studies, 12 studies reported the safety of MBE. Adverse reactions data were not available from five trials (Yang et al., 2005; Teut et al., 2016; Cao, 2018; Qiu, 2018; Liu et al., 2019). Among the studied reporting adverse events, 10 reports (83%) mentioned that there were no adverse events related to MBE intervention. One study (von Trott et al., 2009) reported five minor side effects in qigong group (i.e., two nausea cases, two aching muscles, and one muscle tension). Another study (Raub, 2002) claimed that there was serious adverse event requiring hospitalization in 11 patients, but none of these events was related to the TC. Interventions such as TC, yoga, and qigong were highly safe and tolerable for the elderly who suffering from chronic pain.

## Discussion

### Main Findings

This meta-analysis is the first to explore the effectiveness of three popular movement-based MBE modes systematically (i.e., TC, yoga, and qigong) regarding middle-aged and elderly people with chronic pain throughout comprehensive evaluate current evidence. The total effect amount showed that compared with both the nonactive and active control, MBE >8 sessions could significantly reduce the pain symptoms of middle-aged and elderly people, while the effect amount from large to small was yoga, TC, and qigong. MBE have shown good analgesic effects in the elderly with KOA and CLBP. Moreover, the recent meta-analysis (Morone and Greco, 2007; Chen et al., 2016; Zhang et al., 2017) results of separately summarizing KOA also demonstrated that TC and qigong have a good pain relief effect. However, due to the small sample size and high heterogeneity of CLBP, this effect needed to be carefully explained.

In terms of self-reported function, MBE had obvious benefits for KOA and neck pain, but the efficacy of CLBP was uncertain. TC and qigong improved the function significantly better than the control group, and the results revealed that MBE for at least 30–45 sessions had obvious benefits on the function. The meta of nonspecific LBP in the elderly published by Paulo R.C. found that there was weak and not clinically relevant evidence that yoga had an effect on pain and function (Nascimento et al., 2019). In another study (Wieland et al., 2017), yoga can improve the back-related function and reduce pain in adults with CLBP; however, the ES did not meet predefined levels of minimum clinical importance. The results of the subgroup analysis showed that yoga intervention may not improve the level of function for middle-aged and old patients with chronic pain, which may relate to its complexity compared with the other two MBE modes. Yoga usually requires professional guidance from a certified instructor, and self-practice at home may contribute to wrong movement patterns, which is an important factor affecting function (Sherman et al., 2011; Nambi et al., 2014; Saper et al., 2017). Only three of all included studies involved yoga intervention (Cheung et al., 2014, 2017; Teut et al., 2016), wherein the insufficient power of sample size may have affected the power of detecting significant differences in the level of function.

At present, there was no evidence that MBE has the advantage of improving the QOL. The effect of any type of MBE on MCS in elderly patients with chronic pain was not better than that in the control group. Similarly, MBE has no significant effect on PCS. The results of this study were also consistent with the previous results of MBE application in KOA (Zhang et al., 2017). In addition, Irwin et al. conducted TC intervention on older patients who had suffered chronic pain from the herpes zoster virus and found no significant increase in SF-36 scores (Irwin et al., 2003). One possible explanation for this result relates to the sessions of the MBE intervention and follow-up. MBE may indirectly improve QOL by regulating systemic function, the significant effect of which may take a long time to observe. Only data immediately after intervention were used as outcomes in this meta-analysis, which may lack evidence of QOL during long-term follow-up. It was worth emphasizing that no serious adverse events were found to be associated with MBE.

### Potential Advantages of MBE for Elderly

In addition to reducing pain in elderly patients effectively, TC, as an effective alternative medical means, has been clinically proven to improve self-efficacy and physical function in elderly patients, reduce the risk of falls, and have a positive effect on blood pressure control. TC was also integrated into cognitive behavioral therapy to relieve the pressure of people with human immunodeficiency virus infection (McCain et al., 2008) and also incorporated into the health management of fibromyalgia patients (Wang et al., 2010b). Evidence of chronic pain population suggests that TC training is beneficial to chronic KOA pain and CLBP (Kong et al., 2016; Hall et al., 2017). Our study produced similar results and made up for the limitation of insufficient sample size of these meta-analyses.

A meta-study (Bai et al., 2015) involving 10 RCTs revealed that only internal qigong can improve chronic pain in adults, but external qigong had no significant difference. However, studies on traditional Chinese qigong, such as Wuqinxi and Baduanjin, have not been included in it. The scarcity of trials related to qigong in the English-language articles in contrast with the greater number of TC studies probably indicates the differing popularity of these interventions for the moment in the Western countries. These exercises combine low-effect control exercises, breathing, and meditative awareness and are worth studying in older adults with chronic pain. Although the mechanism is still unclear, qigong exercises can be used as a rehabilitation method to improve the symptoms of chronic pain patients and prevent further deterioration of the disease.

There have been few studies on yoga as an intervention for chronic pain in the elderly. The meta (Büssing et al., 2012) also showed short-term yoga interventions may be effective and could ameliorate several pain-associated disability. Studies on yoga for CLBP have shown that while yoga can reduce pain, there was no significant improvement in QOL or functioning (Teut et al., 2016). In addition, some other trials (Bellamy et al., 1988; Garfinkel et al., 1994; Kolasinski et al., 2005) have also shown that yoga can significantly improve the pain of knee, hip, and hand arthritis in older adults and also presented compelling evidence that yoga is safe.

### Potential Analgesic Mechanism of MBE

The potential mechanisms by which MBE affects pain perception and function levels in older patients with chronic pain were not fully understood. The potential effect of it probably be ascribed these exercises to impact altered central elements such as central pain sensitization. Unlike regular aerobic exercise, MBE emphasizes slow, controlled body movements while regulating focus and awareness through breathing and meditation. Studies have shown that long-term TC practice can induce regional structural changes in the precentral gyrus, insular sulcus, and middle frontal sulcus (Wei et al., 2013). Shen et al. investigated the association between neurobiological effects and pain/physical function among postmenopausal women with KOA after 8 weeks of TC interventions. Moderate-high correlations were observed between TC-associated pre-post changes in amygdala-medial prefrontal cortex functional group connectivity and pain and physical function improvement (Shen et al., 2021), indicating that MBE may directly affect the cerebral cortex to regulate pain and physical function through regular practice.

The effect of MBE may also involve the hypothalamic-pituitary-adrenal (HPA) axis, which dominates the endocrine regulatory system. Under normal conditions, activated HPA axis induced by stress releases cortisol, resulting in the downregulation of inflammatory cells. However, in patients with chronic pain, the existence of pain stress would lead to the disorder of endocrine system. Based on the Feng's view, slow and mild movements of MBE with deep breathing may alter the sympathetic-adrenal-medullary axis (SAM axis; sympathetic nervous system), reducing the HPA axis reactivity (Feng et al., 2020). The mechanism of chronic pain is full of complex factors (Edwards et al., 2006; Campbell and Edwards, 2009; Niederstrasser et al., 2014), which may involve proinflammatory immune responses, stress, indices of central sensitization, and central nociceptive processing system. The levels of stress-related pain and function may be consequently decreased through this HPA mechanism (Thayer and Lane, 2000).

### Safety and Popularization

Extensive evidence not only proved the positive effects of MBE on pain, psychosocial health, stress, anxiety, and depression but also showed that MBE is a suitable and safe exercise pattern for middle-aged and elderly individuals and those who are less likely to engage in intense exercises (Chow and Tsang, 2007; Ross and Thomas, 2010; Wang et al., 2010a). MBE is usually conducted in group classes, which is a typical exercise group or classroom intervention. Therefore, although exercising in various modes, the results may be similar. In addition, there may be some specific scientific effects because a person's interest and enjoyment in a particular activity are often key factors in their adherence to regular practice (Terjestam et al., 2010). At the same time, our review also emphasized the importance of the frequency and duration of MBE interventions, some of which lasted up to 60 min/session. The authors of a study of frail elderly people in nursing homes noted the importance of keeping treatment short (Mcbee et al., 2004). The proposed changes seem reasonable and appropriate to maximize safety and cognitive understanding of therapy. In addition, many studies used the combination of group classes and homework for intervention. The low efficiency of homework exercise may also be an important factor affecting the outcome and reducing the ability to detect the treatment effect.

### Future Directions

The importance of high-quality designing clinical trials targeting older adults needs to be considered for future researchers. Future studies could examine the possible long-term outcomes modifiers present in this population, thus allowing the recommendation of more efficacious evidence-based interventions to this growing population. Additional information needs to be provided on the comparison of symptom improvement in chronic pain patients with various MBE modes and different age groups, such as focus only on elderly patients with chronic pain, which might be worth. More multiarm RCTs should be conducted in the future to determine the efficacy differences of different MBE modes in middle-aged and elderly patients with chronic pain.

### Strengths and Limitations

This study has several strengths as follows:

· We conducted a robust systematic review and meta-analysis regarding the effect of MBE on chronic pain symptoms for middle-aged and elderly people.
· Three popular MBE modes (i.e., TC, yoga, and qigong) are included in the study;
· We included Chinese-language studies and gray literatures, which made the review more comprehensive;
· We used the rigorous meta-analytical ways and assessment, such as sensitivity analyses, additional models using fixed effects, assessment of publication bias, trim and fill analysis, and GRADE approach.

Several limitations should be considered as follows:

· It was difficult to blind subjects and coaches during MBE interventions, which may contribute to potential risks of performance bias. However, this is an inherent limitation of such studies and is usually reported in meta-analyses of exercise interventions (Kong et al., 2016; Goh et al., 2019; Owen et al., 2020). We expect future RCTs to identify appropriate blinding methods for participants and instructors to reduce the risk of performance bias;
· Several studies did not report “random sequence generation,” “intention to treat analysis,” and “allocation concealment,” which probably overstate the pooled ES. However, subsequent sensitivity analysis showed that our results were robust;
· Potential publication bias and heterogeneity of the results were may be influenced by the styles of MBE and chronic pain conditions. This may require more sophisticated analytical methods and separate reporting.

## Conclusion

Our results indicated that MBE (i.e., TC, yoga, and qigong) were effective treatments for reducing symptoms of middle-aged and elderly people with chronic pain compared with the nonactive and active control groups. TC and qigong had obvious benefits for KOA in self-reported function, but the efficacy of CLBP was uncertain. No significant benefit of MBE on QOL in older adults with chronic pain was found. The potential of yoga and TC as two common nonpharmacological treatments for chronic pain needs to be rigorously assessed in future studies. More high-quality RCTs should be conducted to explore the efficacy and mechanism of MBE on chronic pain in middle-aged and elderly people from various dimensions, such as affective and cognitive dimensions.

## Data Availability Statement

The original contributions presented in the study are included in the article/Supplementary Material, further inquiries can be directed to the corresponding authors.

## Author Contributions

Y-RW and JS wrote the manuscript and involved in the data analysis. Y-FW, Y-RW, X-QW, and Y-LW contributed to the conception. Z-YH and JS searched the literature. Y-YL, JS, Y-RW, Y-TL, and Y-LW contributed to the acquisition of data. All authors contributed to the article and approved the submitted version.

## Funding

This study was supported by the grant from the National Key R&D Program of China (2020YFC2007700) and the Guangdong Hopson-Pearl River Education Development Foundation (Grant No. H20190116202012724).

## Conflict of Interest

The authors declare that the research was conducted in the absence of any commercial or financial relationships that could be construed as a potential conflict of interest.

## Publisher's Note

All claims expressed in this article are solely those of the authors and do not necessarily represent those of their affiliated organizations, or those of the publisher, the editors and the reviewers. Any product that may be evaluated in this article, or claim that may be made by its manufacturer, is not guaranteed or endorsed by the publisher.
