# Supplementary material for: Are Mind-Body Exercise Beneficial for Treating Pain, Function, and Quality of Life in Middle-Aged and Old People With Chronic Pain? A Systematic Review and Meta-Analysis
Source: Front Aging Neurosci. 2022 Jun 21;14:921069. doi: 10.3389/fnagi.2022.921069 (PMC9255956; doi:10.3389/fnagi.2022.921069)
Supplement: Supplementary file 2 [file Data_Sheet_2.docx]

**PubMed**

#1 “Yoga”[MeSH Terms]OR”yoga” OR ”hatha yoga” OR “hot yoga” OR “iyengar yoga” OR “pranayama” OR “yoga nidra”

#2 “tai ji"OR "Tai Chi" OR "chi tai"OR "Tai Ji Quan"OR "ji quan tai"OR "quan tai ji" OR "Taiji" OR "Taijiquan" OR "T'ai Chi" OR "Tai Chi Chuan" OR "tai ji"[MeSH Terms]

#3 "qigong"[MeSH Terms] OR “Qi Gong” OR “Ch'i Kung” OR “baduanjin” OR “yijinjing” OR “liuzijue” OR “wuqinxi”

#4 “mind-body” OR “mind exercise”

#5: #1 OR #2 OR #3 OR #4

#6: ("Chronic Pain"[Mesh]) OR (Chronic Pains) OR (Widespread Chronic Pain)

#7: (sciatica OR back‐ache OR back* ache OR lumbago OR fibromyalg* OR "reflex dystroph*" OR "sudeck* atroph*" OR causalg* OR whip‐lash OR whip* lash OR polymyalg* OR "failed back surg*" OR "failed back syndrome*") OR ((tempromandib* joint*) AND (pain*)) OR ((temperomandib* joint*) AND (pain*)) OR ((temporomandib* joint*) AND (pain*)) OR ((chronic* OR back OR musculoskel* OR intractabl* OR neuropath* OR phantom limb OR fantom limb OR neck OR myofasc* OR central OR post* stroke OR complex OR regional OR spinal cord) AND pain*))

#8: #6OR#7

#9: #8AND#5

#10: aged OR aging OR old∗ OR elder∗ OR senior∗ OR “middle aged” OR"Aged"[Mesh]

#11: #9 AND #10

**Cochrane**

#1 MeSH descriptor: [Tai Ji] explode all trees

#2 ("tai ji" OR "Tai Chi" OR "chi tai" OR "Tai Ji Quan" OR "ji quan tai" OR "quan tai ji" OR Taiji OR "Taijiquan" OR "T'ai Chi" OR "Tai Chi Chuan"):ti,ab,kw

#3: #1 OR #2

#4: MeSH descriptor: [Qigong] explode all trees

#5: ("qigong" OR "Qi Gong" OR "Ch'i Kung" OR "baduanjin" OR "yijinjing" OR "liuzijue" OR "wuqinxi"):ti,ab,kw

#6: #4 OR #5

#7: MeSH descriptor: [Yoga] explode all trees

#8: ("Yoga" OR "hatha yoga" OR "hot yoga" OR "iyengar yoga" OR "pranayama" OR "yoga nidra"):ti,ab,kw

#9: #7OR#8

#10: (“mind-body exercise”):ti,ab,kw

#11: #3OR#6OR#9OR#10

#12: MeSH descriptor: [Chronic pain] explode all trees

#13: (chronic* or back or musculoskel* or intractabl* or neuropath* or phantom limb or fantom limb or neck or myofasc* or "temporomandib* joint" or "temperomandib* joint" or "tempromandib* joint" or central or (post next stroke) or complex or regional or "spinal cord") near/4 pain*:ti,ab,kw (Word variations have been searched)

#14: (sciatica or back‐ache or back*ache or lumbago or fibromyalg* or (trigemin* near/2 neuralg*) or (herp* near/2 neuralg*) or (diabet* near/2 neuropath*) or (reflex near/4 dystroph*) or (sudeck* near/2 atroph*) or causalg* or whip‐lash or whip*lash or polymyalg* or (failed back near/4 surg*) or (failed back near/4 syndrome*)):ti,ab,kw (Word variations have been searched)

#15: #12 OR #13 OR #14

#16: MeSH descriptor: [Aged] explode all trees

#17: ((aged OR aging OR old∗ OR elder∗ OR senior∗ OR “middle age”)):ti,ab,kw

#18: #16OR#17

#19: #11AND#15AND#18

**Embase**

#1: 'Tai Chi'/exp

#2: 'taiji quan':ti,ab,kw OR taijiquan:ti,ab,kw OR taiji:ti,ab,kw OR 'tai ji':ti,ab,kw OR 'tai chi':ti,ab,kw OR 'chi tai':ti,ab,kw

#3: 'qigong'/exp

#4: qigong:ti,ab,kw OR 'qi gong':ti,ab,kw OR qi‐gong:ti,ab,kw OR 'qi kung':ti,ab,kw OR 'jhi gong':ti,ab,kw OR 'chi gung':ti,ab,kw OR 'qi chung':ti,ab,kw OR 'chi kung':ti,ab,kw OR 'kung chi':ti,ab,kw OR baduanjin:ti,ab,kw OR wuqinxi:ti,ab,kw OR liuzijue:ti,ab,kw OR yijinjing:ti,ab,kw

#5: 'yoga'/exp

#6: yoga:ti,ab,kw OR 'hatha yoga':ti,ab,kw OR 'hot yoga':ti,ab,kw OR 'iyengar yoga':ti,ab,kw OR pranayama:ti,ab,kw OR 'yoga nidra':ti,ab,kw

#7: 'mind -body exercise':ti,ab,kw OR 'mindful body':ti,ab,kw

#8: #1OR#2OR#3OR#4OR#5OR#6OR#7

#9: 'chronic pain'/exp

#10: 'chronic pains':ti,ab,kw OR 'widespread chronic pain':ti,ab,kw OR 'widespread chronic pains':ti,ab,kw OR sciatica:ti,ab,kw OR back‐ache:ti,ab,kw OR 'back* ache':ti,ab,kw OR lumbago:ti,ab,kw OR fibromyalg*:ti,ab,kw OR 'reflex dystroph*':ti,ab,kw OR 'sudeck* atroph*':ti,ab,kw OR causalg*:ti,ab,kw OR whip‐lash:ti,ab,kw OR 'whip* lash':ti,ab,kw OR polymyalg*:ti,ab,kw OR 'failed back surg*':ti,ab,kw OR 'failed back syndrome*':ti,ab,kw OR ('tempromandib* joint*':ti,ab,kw AND pain*:ti,ab,kw) OR ('temperomandib* joint*':ti,ab,kw AND pain*:ti,ab,kw) OR ('temporomandib* joint*':ti,ab,kw AND pain*:ti,ab,kw) OR ((chronic*:ti,ab,kw OR back:ti,ab,kw OR musculoskel*:ti,ab,kw OR intractabl*:ti,ab,kw OR neuropath*:ti,ab,kw OR 'phantom limb':ti,ab,kw OR 'fantom limb':ti,ab,kw OR neck:ti,ab,kw OR myofasc*:ti,ab,kw OR central:ti,ab,kw OR 'post* stroke':ti,ab,kw OR complex:ti,ab,kw OR regional:ti,ab,kw OR 'spinal cord':ti,ab,kw) AND pain*:ti,ab,kw)

#11: #9OR#10

#12: 'aged'/exp

#13: aged:ti,ab,kw OR aging:ti,ab,kw OR old∗:ti,ab,kw OR elder∗:ti,ab,kw OR senior∗:ti,ab,kw OR 'middle age':ti,ab,kw

#14: #12OR#13

#15: #8AND#11AND#14

**WOS**

#1 “Yoga” OR “hatha yoga” OR “hot yoga” OR “iyengar yoga” OR “pranayama” OR “yoga nidra”

#2 "tai ji" OR "Tai Chi" OR "chi tai" OR "Tai Ji Quan" OR "ji quan tai" OR "quan tai ji" OR "Taiji" OR "Taijiquan" OR "T'ai Chi" OR "Tai Chi Chuan"

#3 "qigong" OR “Qi Gong” OR “Ch'i Kung” OR “baduanjin” OR “yijinjing” OR “liuzijue” OR”wuqinxi”

#4 “mind-body” OR “mind exercise”

#5: #1OR#2OR#3#OR#4

#6: ((Chronic Pain) OR (Chronic Pains) OR (Widespread Chronic Pain) OR (Widespread Chronic Pains) OR (sciatica OR back‐ache OR back* ache OR lumbago OR fibromyalg* OR "reflex dystroph*" OR "sudeck* atroph*" OR causalg* OR whip‐lash OR whip* lash OR polymyalg* OR "failed back surg*" OR "failed back syndrome*") OR ((tempromandib* joint*) AND (pain*) ) OR ((temperomandib* joint*) AND (pain*) )) OR ((temporomandib* joint*) AND (pain*))OR ((chronic* OR back OR musculoskel* OR intractabl* OR neuropath* OR phantom limb OR fantom limb OR neck OR myofasc* OR central OR post* stroke OR complex OR regional OR spinal cord) AND (pain*))

#7: aged OR aging OR old∗ OR elder∗ OR senior∗ OR “middle aged”

#8: #5AND#6AND#7

**Chinese database**

#1: 太极(tai chi)

#2: 太极拳(taijiquan)

#3: 气功(qigong)

#4: 八段锦(baduanjin)

#5: 五禽戏(wuqinxi)

#6: 六字诀(liuzijue)

#7: 易筋经(yijinjing)

#8: 瑜伽(yoga)

#9: 身心运动(mind-body exercise)

#10: #1OR#2OR#3OR#4OR#5OR#6OR#7OR#8OR#9

#11: 慢性疼痛(chronic pain)

#12: 慢性腰痛(chronic low back pain)

#13: 骨关节炎(osteoarthritis)

#14: 颈痛(neck pain)

#15: 神经病理性疼痛(neuropathic pain)

#16: 中风后疼痛(Central post-stroke pain)

#17：#11OR#12OR#13OR#14OR#15OR#16

#18: 中老年人(middle-aged and old people)

#19: 老年人(old people)

#20: #18OR#19

#21: #10AND#17AND#20
