# Supplementary material for: Are Mind-Body Exercise Beneficial for Treating Pain, Function, and Quality of Life in Middle-Aged and Old People With Chronic Pain? A Systematic Review and Meta-Analysis
Source: Front Aging Neurosci. 2022 Jun 21;14:921069. doi: 10.3389/fnagi.2022.921069 (PMC9255956; doi:10.3389/fnagi.2022.921069)
Supplement: Supplementary file 3 [file Data_Sheet_3.docx]

| **MBEs group compared to control group for middle-aged and elderly people with chronic pain conditions** | | | | | | |
| --- | --- | --- | --- | --- | --- | --- |
| **Patient or population:** patients with middle-aged and elderly people with chronic pain conditions **Settings:**  **Intervention:** MBEs group **Comparison:** control group | | | | | | |
| **Outcomes** | **Illustrative comparative risks* (95% CI)** | | **Relative effect (95% CI)** | **No of Participants (studies)** | **Quality of the evidence (GRADE)** | **Comments** |
|  | Assumed risk | Corresponding risk |  |  |  |  |
|  | **Control group** | **MBEs group** |  |  |  |  |
| **Pain intensity** |  | The mean pain in the intervention groups was **0.64 standard deviations lower** (0.86 to 0.42 lower) |  | 1332 (17 studies) | ⊕⊝⊝⊝ **very low**^1^ | SMD -0.64 (-0.86 to -0.42) |
| **Physical Function** |  | The mean function in the intervention groups was **0.75 standard deviations lower** (1.13 to 0.37 lower) |  | 1082 (12 studies) | ⊕⊝⊝⊝ **very low**^1^ | SMD -0.75 (-1.13 to -0.37) |
| **QOL(MCS)** |  | The mean QoL (MCS) in the intervention groups was **0.01 standard deviations lower** (0.39 lower to 0.36 higher) |  | 787 (8 studies) | ⊕⊕⊝⊝ **low**^2^ | SMD -0.01 (-0.39 to 0.36) |
| **QOL(PCS)** |  | The mean QoL (PCS) in the intervention groups was **0.23 standard deviations higher** (0.16 lower to 0.62 higher) |  | 787 (8 studies) | ⊕⊕⊝⊝ **low**^2^ | SMD 0.23 (-0.16 to 0.62) |
| *The basis for the **assumed risk** (e.g., the median control group risk across studies) is provided in footnotes. The **corresponding risk** (and its 95% confidence interval) is based on the assumed risk in the comparison group and the **relative effect** of the intervention (and its 95% CI). **CI:** Confidence interval; **QOL:** quality of life; **MCS:** mental component summary; **PCS:** physical component summary. | | | | | | |
| GRADE Working Group grades of evidence **High quality:** Further research is very unlikely to change our confidence in the estimate of effect.  **Moderate quality:** Further research is likely to have an important impact on our confidence in the estimate of effect and may change the estimate. **Low quality:** Further research is very likely to have an important impact on our confidence in the estimate of effect and is likely to change the estimate. **Very low quality:** We are very uncertain about the estimate. | | | | | | |
| ^1^ Downgraded once for study limitations due to high or unclear risk of bias and once for inconsistency due to heterogeneity and once for the asymmetrical distribution of the results in the funnel plot. ^2^ Downgraded once for study limitations due to high or unclear risk of bias and once for inconsistency due to heterogeneity | | | | | | |
