# Supplementary material for: Are Mind-Body Exercise Beneficial for Treating Pain, Function, and Quality of Life in Middle-Aged and Old People With Chronic Pain? A Systematic Review and Meta-Analysis
Source: Front Aging Neurosci. 2022 Jun 21;14:921069. doi: 10.3389/fnagi.2022.921069 (PMC9255956; doi:10.3389/fnagi.2022.921069)
Supplement: Supplementary file 4 [file Data_Sheet_4.docx]

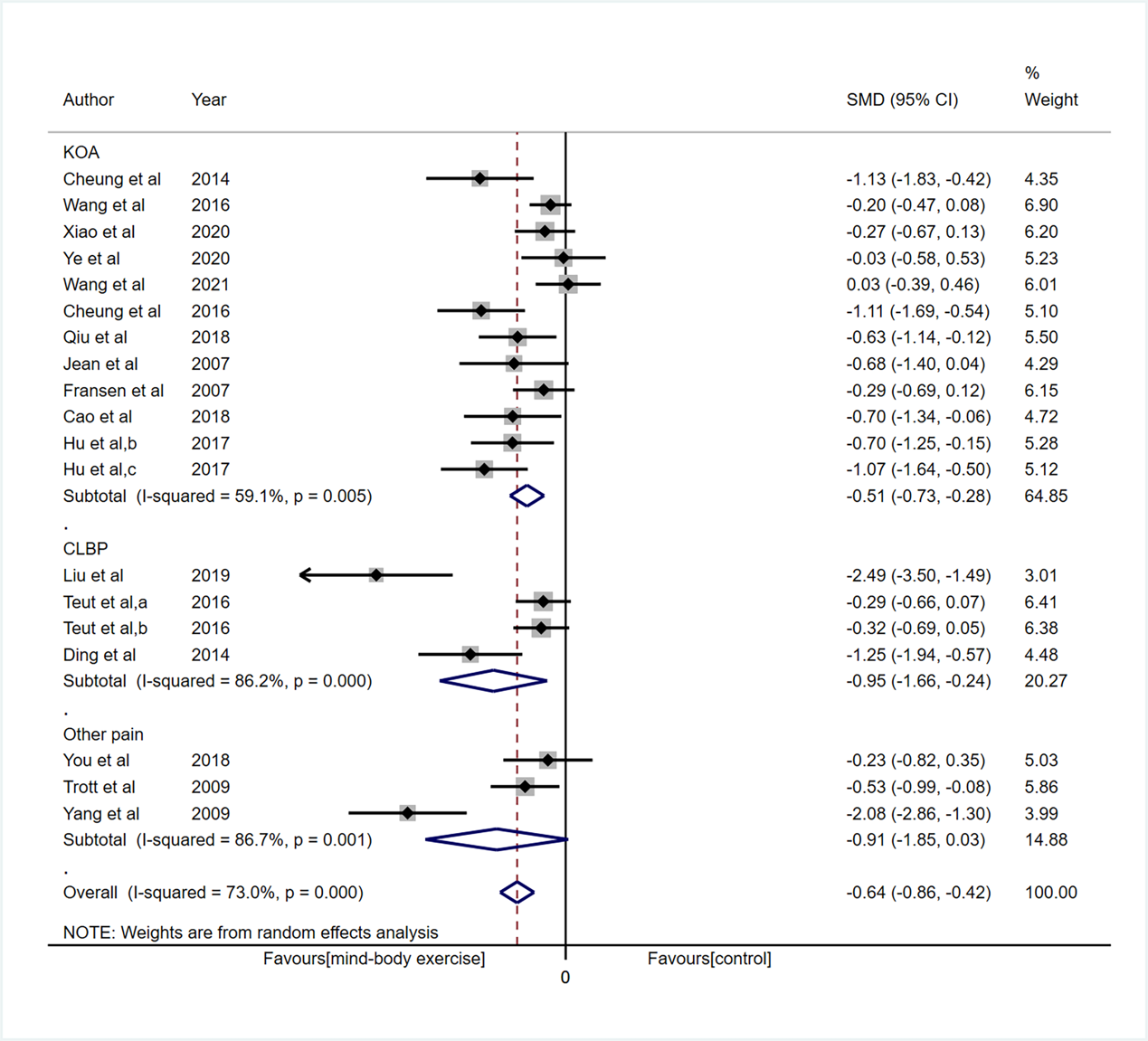


**Supplementary Figure S1.** Forest plot showing SMDs in pain intensity scores from sub-analysis of different chronic pain conditions in middle-aged and elderly patients. NOTE: CI confidence interval, SMD standard mean difference, KOA knee osteoarthritis, CLBP chronic low back pain, a yoga, b tai chi, c qigong.


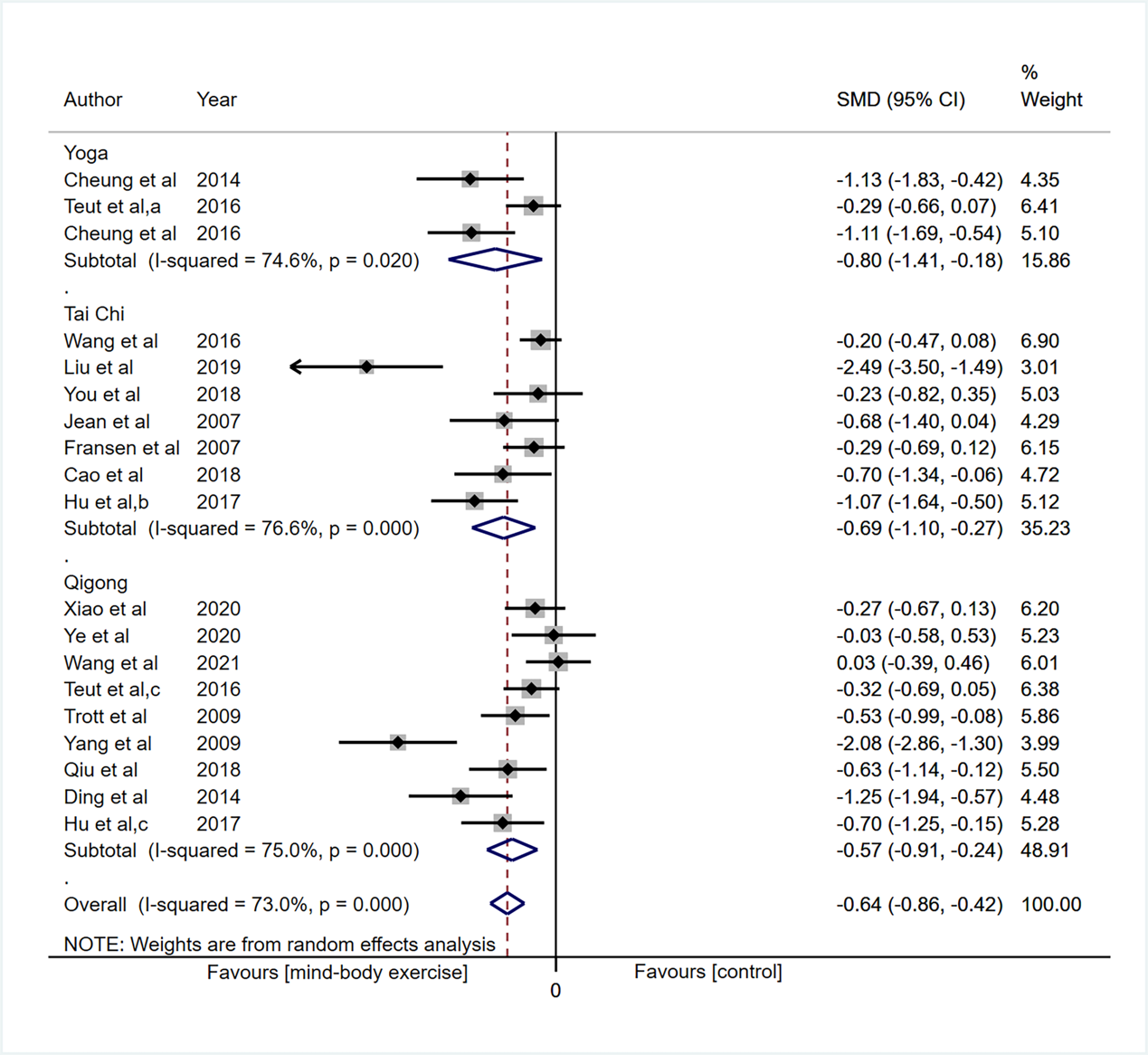


**Supplementary Figure S2.** Forest plot showing SMDs in pain intensity scores from sub-analysis of different types of mind-body exercises. NOTE: CI confidence interval, SMD standard mean difference, a yoga, b tai chi, c qigong.


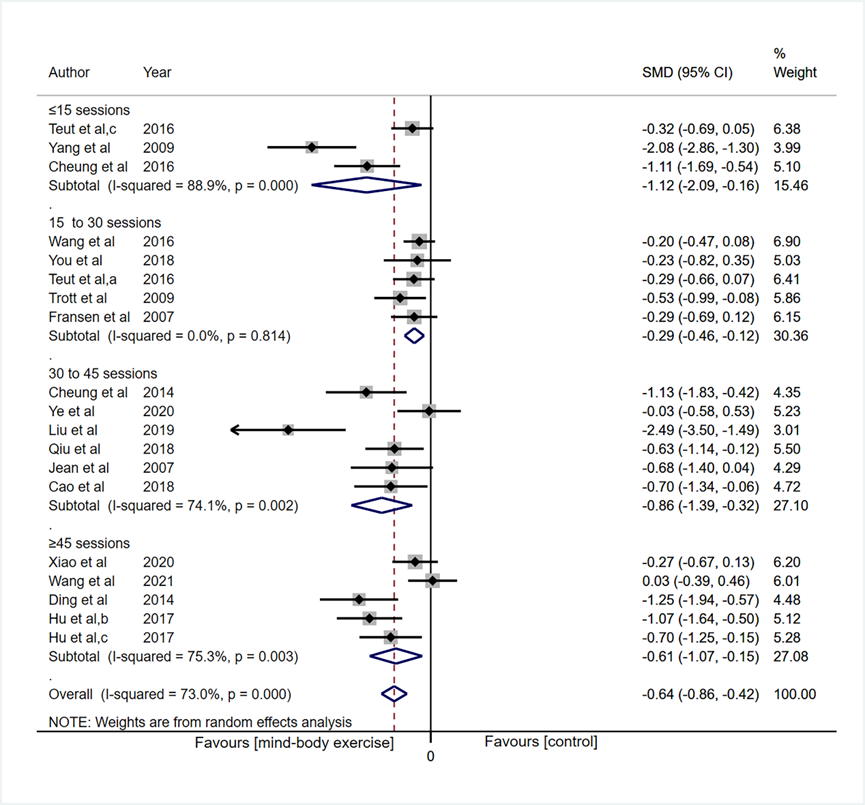


**Supplementary Figure S3.** Forest plot showing SMDs in pain intensity scores from sub-analysis of the number of sessions. NOTE: CI confidence interval, SMD standard

mean difference, a yoga, b tai chi, c qigong.


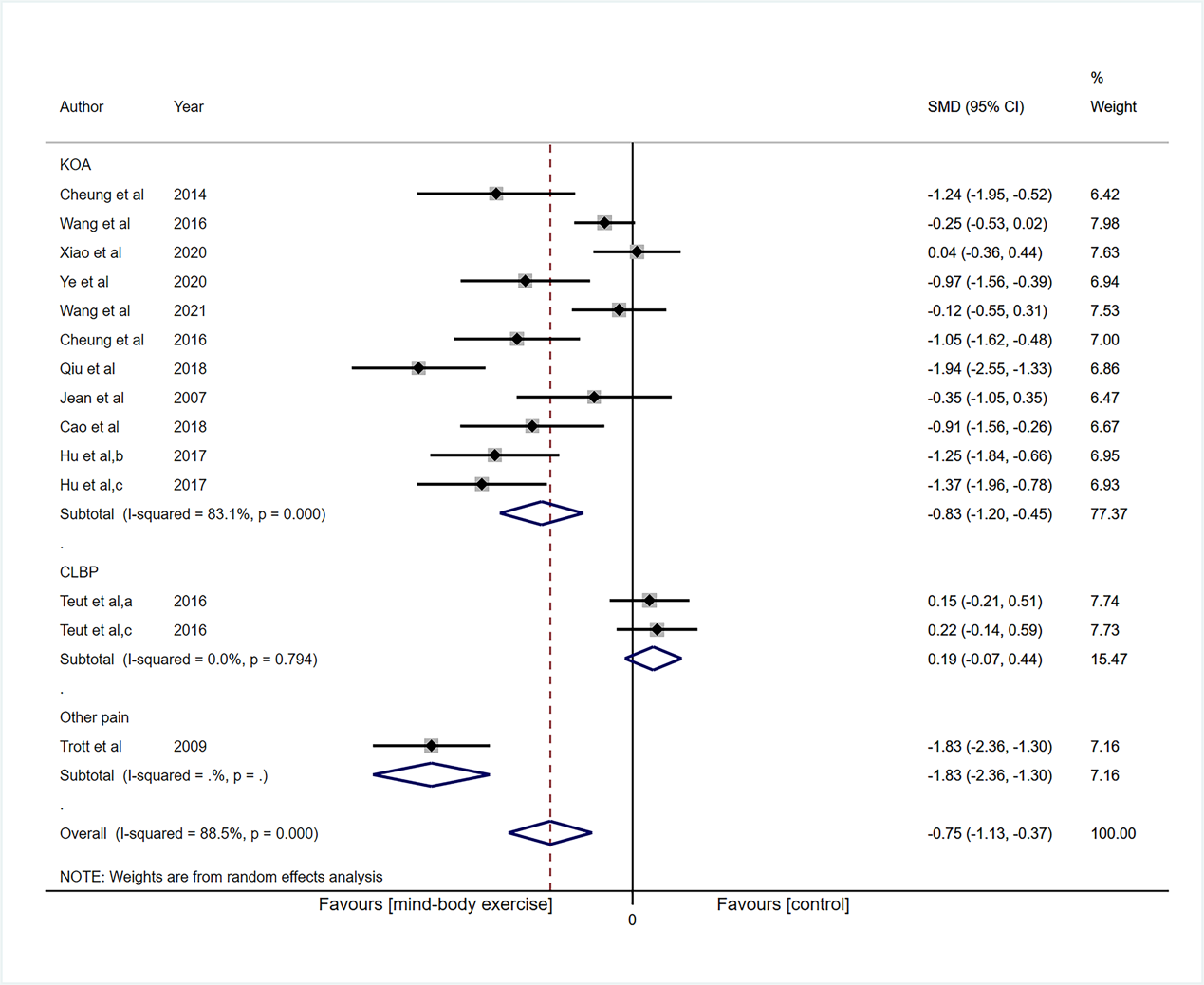


**Supplementary Figure S4.** Forest plot showing SMDs in self-reported function scores from sub-analysis of different chronic pain conditions in middle-aged and elderly patients. NOTE: CI confidence interval, SMD standard mean difference, KOA knee osteoarthritis, CLBP chronic low back pain, a yoga, b tai chi, c qigong.


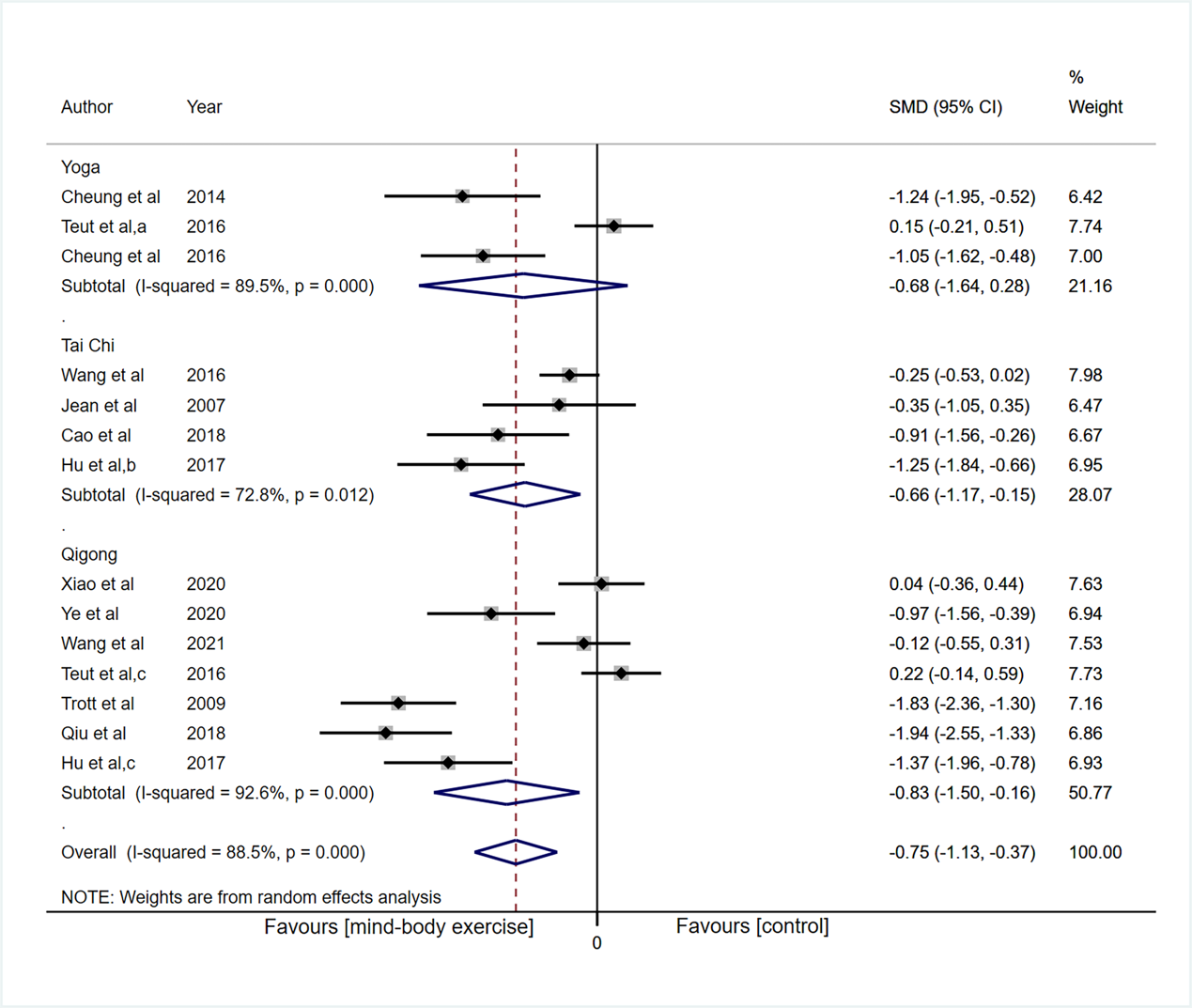


**Supplementary Figure S5.** Forest plot showing SMDs in self-reported function scores from sub-analysis of different types of mind-body exercises. NOTE: CI confidence interval, SMD standard mean difference, a yoga, b tai chi, c qigong.


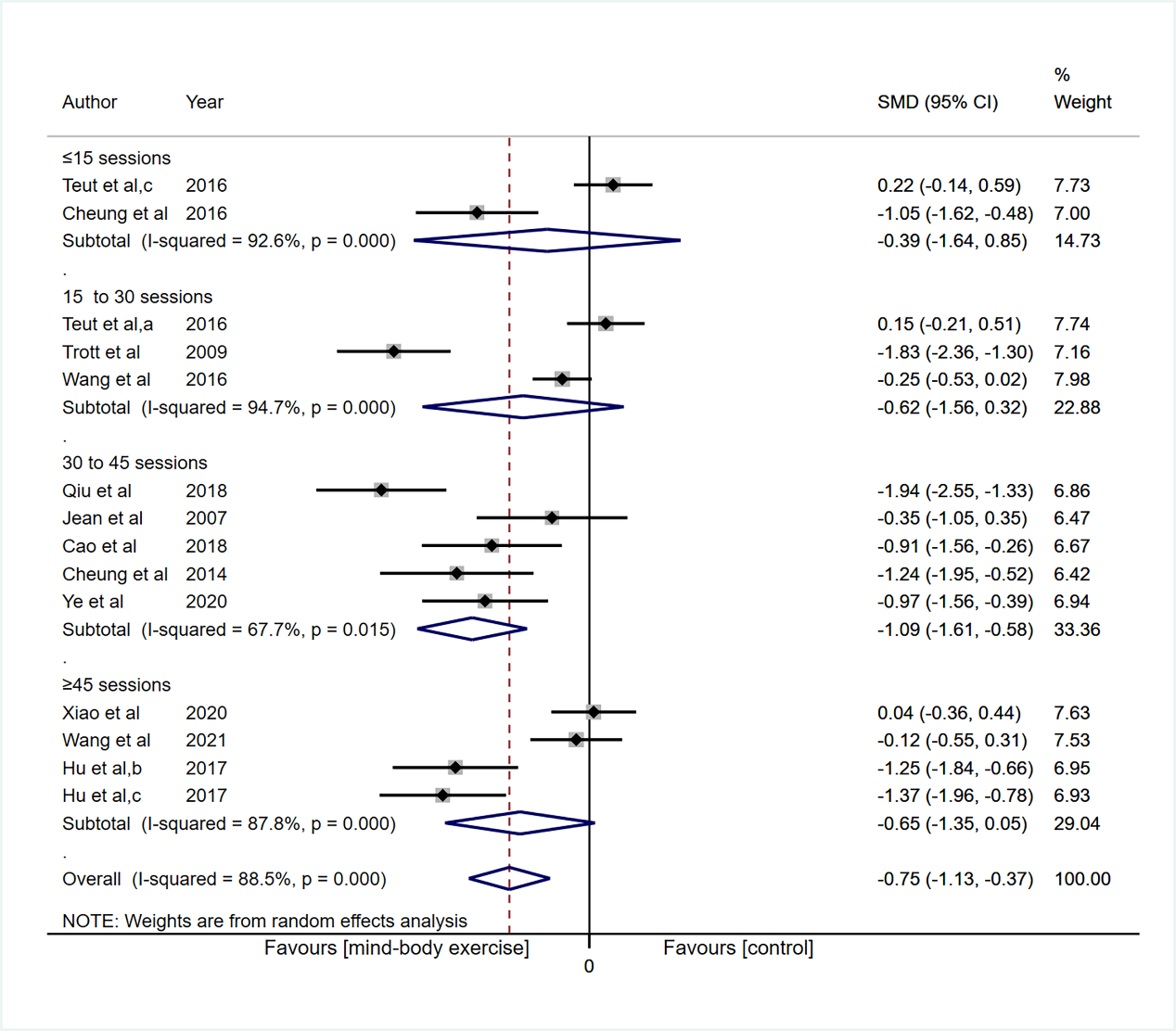


**Supplementary Figure S6.** Forest plot showing SMDs in self-reported function scores from sub-analysis of the number of sessions. NOTE: CI confidence interval, SMD standard mean difference, a yoga, b tai chi, c qigong.


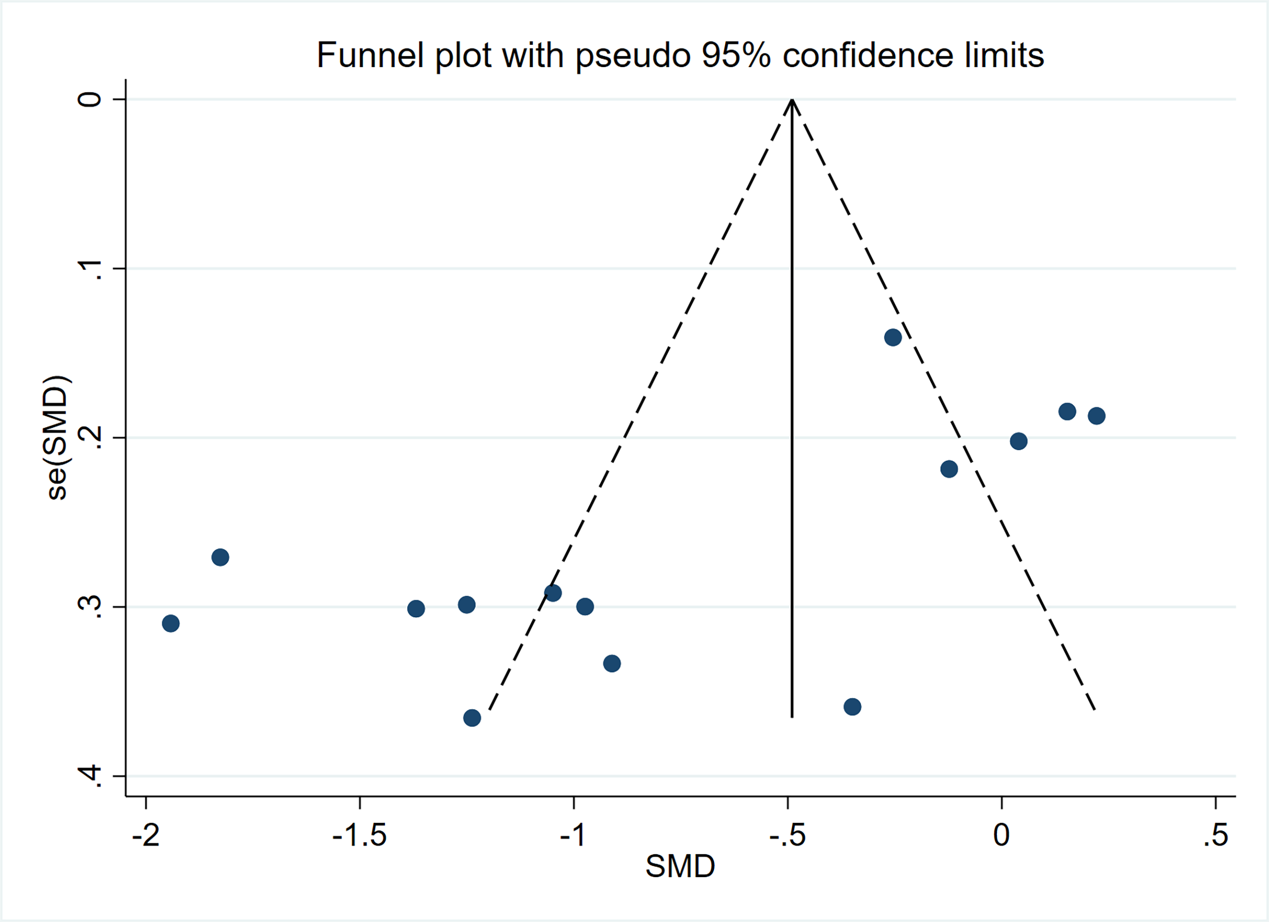


**Supplementary Figure S7.** Funnel plots with standard errors plotted against effect sizes for determining publication bias in self-reported function.
